# Funding Source and Research Report Quality in Nutrition Practice-Related Research

**DOI:** 10.1371/journal.pone.0028437

**Published:** 2011-12-06

**Authors:** Esther F. Myers, J. Scott Parrott, Deborah S. Cummins, Patricia Splett

**Affiliations:** 1 Research and Strategic Business Development, American Dietetic Association, Chicago, Illinois, United States of America; 2 Department of Nutritional Sciences, University of Medicine and Dentistry of New Jersey, Newark, New Jersey, United States of America; 3 Splett and Associates, Stanchfield, Minnesota, United States of America; Copenhagen University Hospital Gentofte, Denmark

## Abstract

**Background:**

The source of funding is one of many possible causes of bias in scientific research. One method of detecting potential for bias is to evaluate the quality of research reports. Research exploring the relationship between funding source and nutrition-related research report quality is limited and in other disciplines the findings are mixed.

**Objective:**

The purpose of this study is to determine whether types of funding sources of nutrition research are associated with differences in research report quality.

**Design:**

A retrospective study of research reporting quality, research design and funding source was conducted on 2539 peer reviewed research articles from the American Dietetic Association's Evidence Analysis Library® database.

**Results:**

Quality rating frequency distributions indicate 43.3% of research reports were rated as positive, 50.1% neutral, and 6.6% as negative. Multinomial logistic regression results showed that while both funding source and type of research design are significant predictors of quality ratings (χ2 = 118.99, p<0.001), the model's usefulness in predicting overall research report quality is little better than chance. Compared to research reports with government funding, those not acknowledging any funding sources, followed by studies with University/hospital funding were more likely to receive neutral vs positive quality ratings, OR = 1.85, *P* <0.001 and OR = 1.54, *P*<0.001, respectively and those that did not report funding were more likely to receive negative quality ratings (OR = 4.97, *P*<0.001). After controlling for research design, industry funded research reports were no more likely to receive a neutral or negative quality rating than those funded by government sources.

**Conclusion:**

Research report quality cannot be accurately predicted from the funding source after controlling for research design. Continued vigilance to evaluate the quality of all research regardless of the funding source and to further understand other factors that affect quality ratings are warranted.

## Introduction

**Figure 1:**
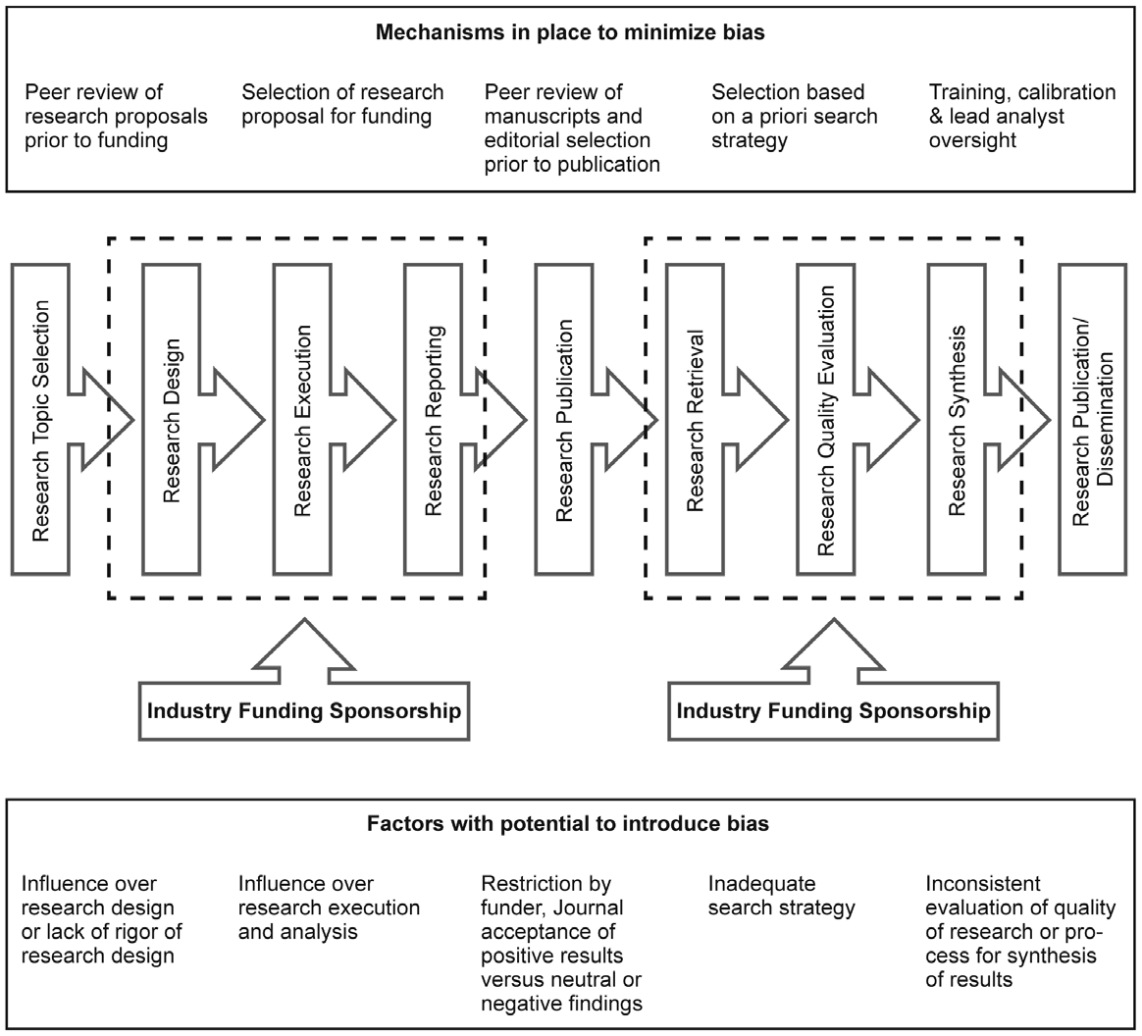
The research process and bias.

The use of the scientific method, traditions of the scientific community, and guidelines for research reporting serve to minimize research bias (defined as systematic deviation of research results or inferences from the truth) by individual or institutional interests [1]. As diagrammed in Figure 1, and reported by several authors, there is potential for bias to enter during the phase of primary research and when published studies are reviewed and synthesized for evidence analysis or review papers [2]–[6]. Studies investigating both non-nutrition-related and nutrition-related research have reported that published findings are likely to favor funder interests [7]–[14]. If this phenomenon were due to researchers with a vested interest in the outcomes of the research being less rigorous in their adherence to standards of execution or reporting of scientific research and thus, consciously or unconsciously skewing their findings in favor of the preferred outcome, it would likely be reflected in lower research report quality ratings when research reports are reviewed and appraised for inclusion in systematic reviews.

Over the past 20 years, a growing amount of research literature has documented concerns regarding the influence of the funding source on research. Government agencies, journal publishers, and other research-focused organizations have developed guidelines for managing potential conflicts of interest or competing interests [15]–[18]. Data are mixed on the relationship between research report quality and the funding source across healthcare disciplines [6], [19]–[22]. Findings indicate that the relationship may vary by the segment of industry or subspecialty (e.g., knee construction, spine, trauma), the type of support (e.g., stock ownership, speaking engagements, or grant receipt), and the type of trial (e.g., drug trial, surgical trial, or other therapies) [7], [10], [11], [23]. Similar data on nutrition-related topics are limited, and some studies have indicated that industry-funded research reports may be of equal or higher quality than non-industry-funded nutrition-related research [24]–[26]. Additional studies are needed to clarify whether the quality of the research report is related to the funding sources in nutrition-related research.

When conducting systematic reviews to provide guidance for clinical practice, research priorities, or to inform public policy, the methodology should yield results that articulate the level of confidence in the outcomes of the research. The Agency for Healthcare Research and Quality (AHRQ) report identified appraising quality of each research report as a key domain in systems that conduct systematic reviews [27].

The recently released Institute Of Medicine report with standards for systematic reviews for comparative effectiveness reviews of medical and surgical interventions identified the following elements for critically appraising each individual study: assessing risk of bias, relevance of the study's population, intervention, outcome measures, and the fidelity of the implementation of interventions [28]. The assessment of these elements is usually based on information in the published research report.

**Figure 2:**
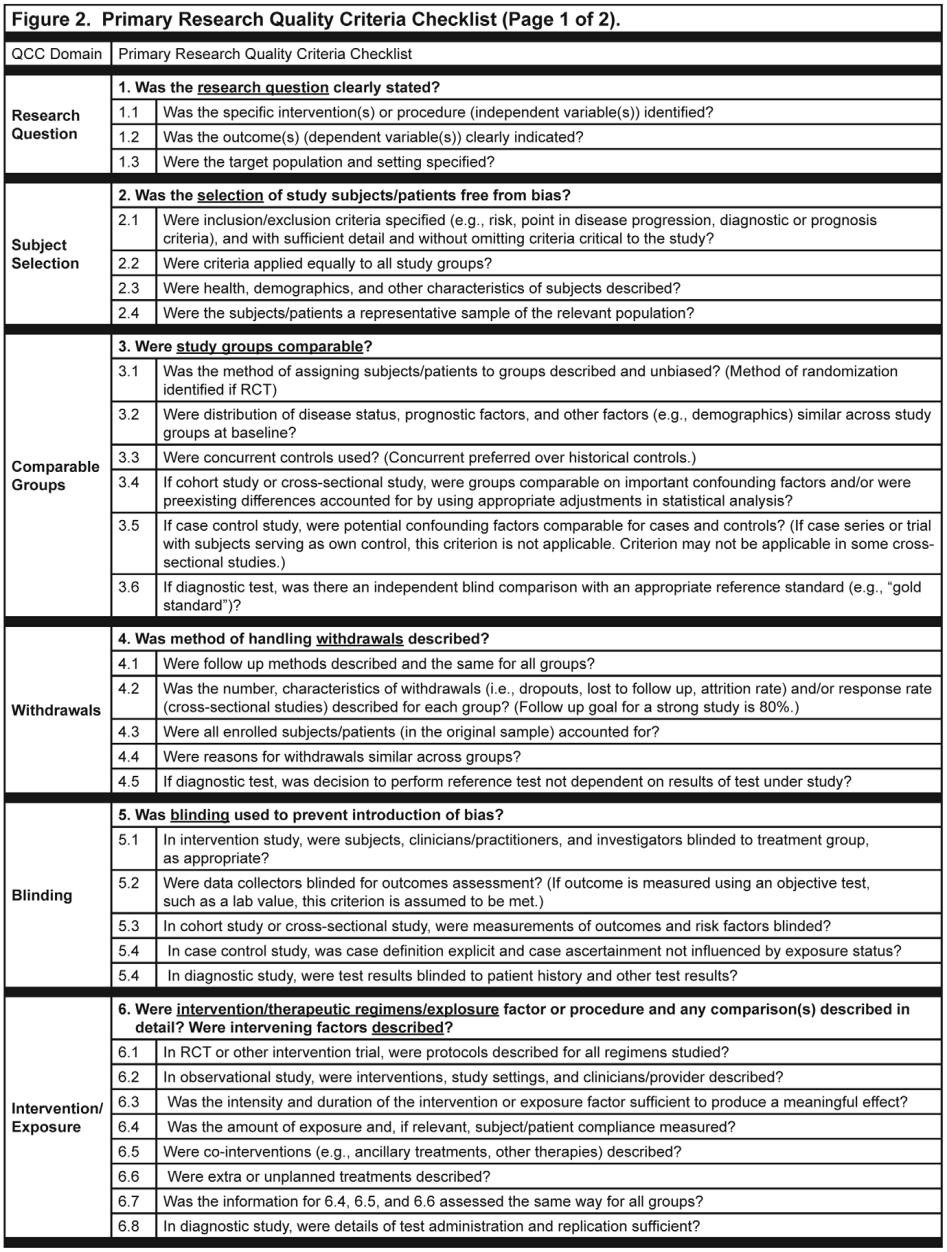
Sample of the American Dietetic Association '**s primary research quality criteria checklist.**

**Figure 3:**
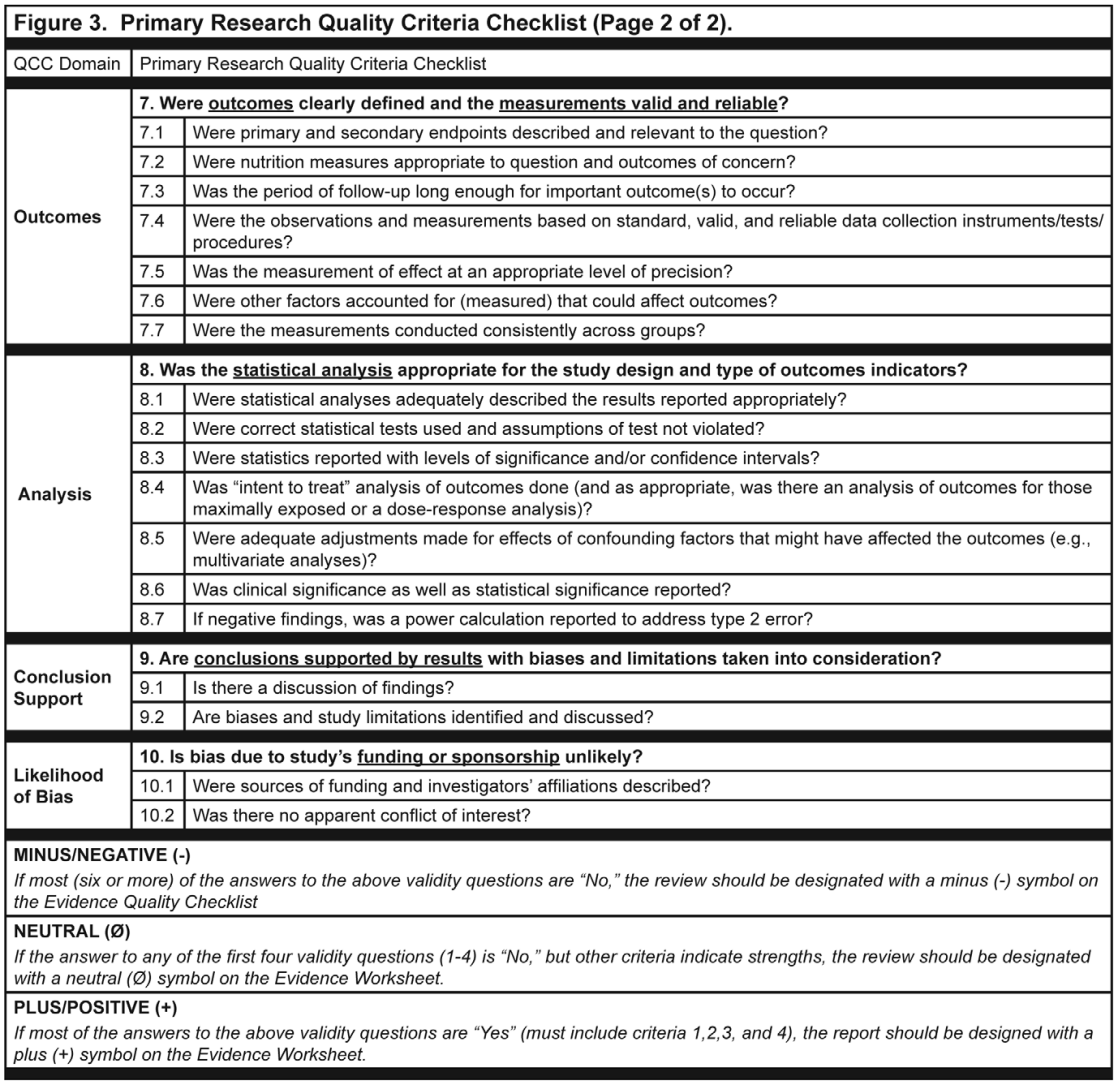
Sample of the American Dietetic Association '**s primary research quality criteria checklist.**

**Figure 4:**
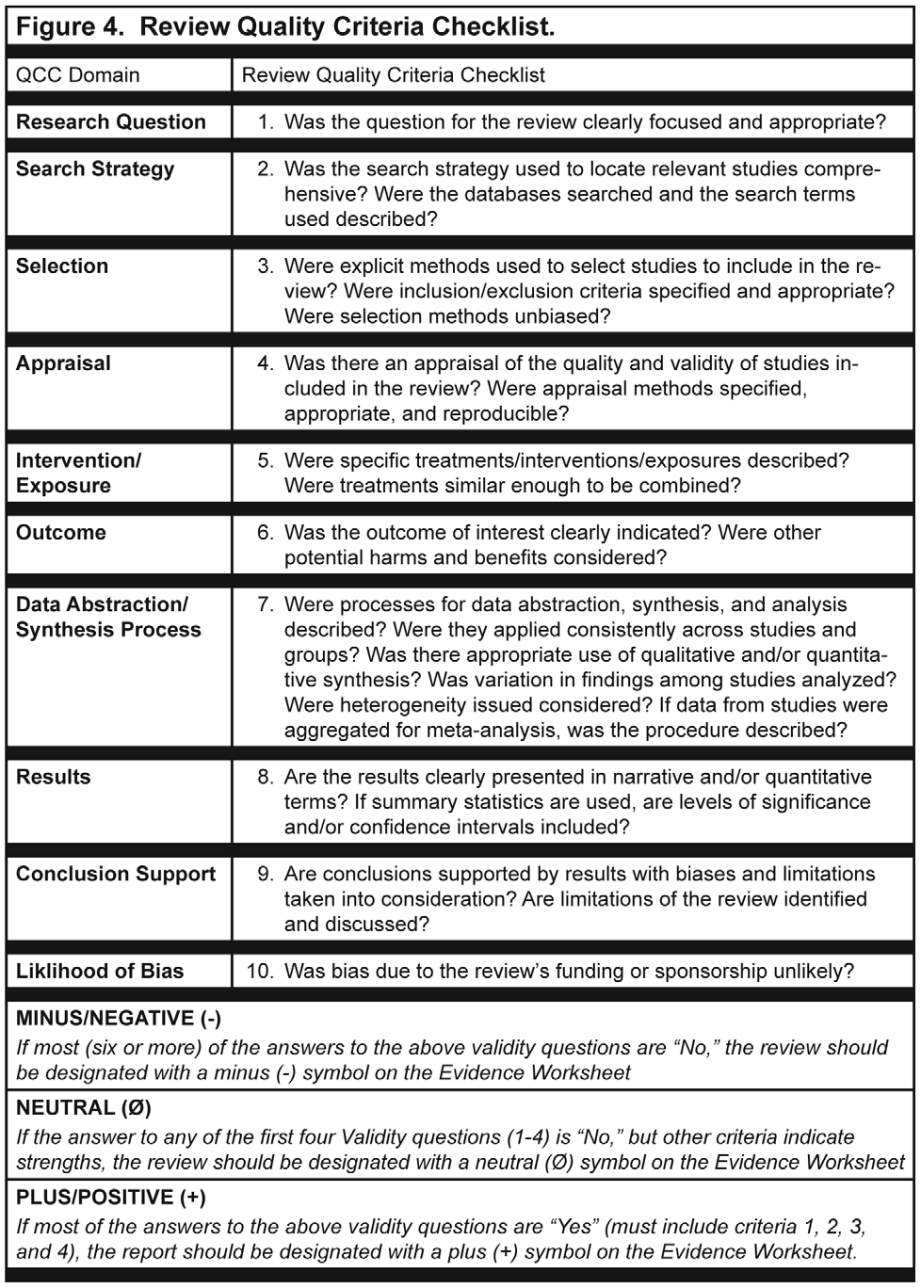
Sample of the American Dietetic Association's review research quality criteria checklist.

A variety of instruments for appraising the quality of research reports have been developed; however, no gold standard has been identified [29]–[31]. For this research, quality of the research report was determined by the presence or absence of threats to validity in the research question, subject selection or search strategy, comparable groups, withdrawals, blinding, appraisal, intervention/exposure, outcomes, analysis or data abstraction/synthesis, conclusion support, and/or likelihood of bias. The Quality Criteria Checklist (QCC), used to evaluate the quality of nutrition-related research reports included in this study had two versions: the Primary Research QCC [Figure 2 and 3] and the Review Research QCC [Figure 4]. Instruments published, in development, or in use before 2009 that were applicable to the topics and research designs included in the American Dietetic Association's (ADA's) Evidence Analysis Library (www.adaevidencelibrary.com) were reviewed. Current editions of Consolidated Standards for Reporting Trials (CONSORT), Strengthening of Reporting of Observational Studies in Epidemiology (STROBE), Transparent Reporting of Evaluations with Nonrandomized Designs (TREND) and the ADA Primary Research QCC were compared [32]–[38]. The following domains were represented in all four instruments: research question, subject selection or search strategy, comparable groups, withdrawals, blinding, intervention/exposure, outcomes, analysis or data abstraction/synthesis, conclusion support, and likelihood of bias. The levels of specificity, organization and interpretation of domain by research design varied among the instruments. Since three of the instruments were specific to a particular research design, the interpretation of the domains varied among the instruments, particularly in the domains for comparable groups, blinding, and intervention/exposure. The Primary Research QCC is a tool with different questions within domains that are applicable to differing research designs.

The Preferred Reporting Items for Systematic Reviews and Meta-Analyses (PRISMA) [updated QUORUM instrument], Meta-analysis of Observational Studies in Epidemiology (MOOSE), A Measurement Tool to Assess Systematic Reviews (AMSTAR) and the ADA Review Research QCC were also compared [38]–[42]. The major domains of research question, subject selection or search strategy, appraisal, intervention/exposure, outcomes, data abstraction/synthesis, results, conclusion support and likelihood of bias were represented in all instruments. There were varying levels of specificity among the instruments within each domain and differing organization of the specific items being rated. The STROBE, MOOSE, and ADA's Review Research QCC were most similar in content.

### Description of Evidence Analysis Process on Nutrition-Related Topics

**Figure 5:**
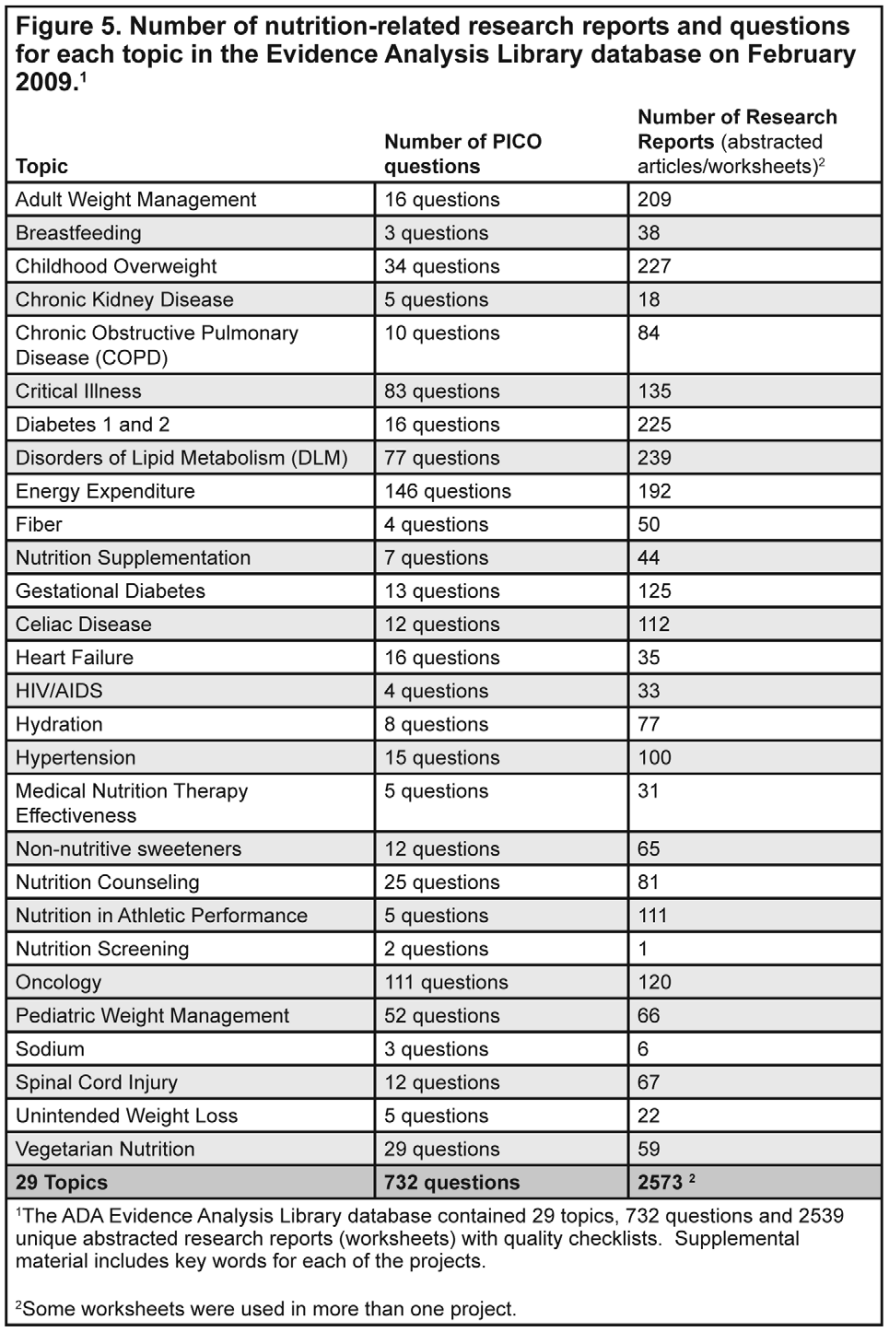
Number of nutrition-related research reports and questions for each topic in the Evidence Analysis Library database on February 2009.

ADA has been conducting systematic reviews for nutrition-related issues since 2000 following a detailed evidence analysis process that includes a quality appraisal of every included research report [38]. QCCs developed for the appraisals were based on the AHRQ criteria [38]. Research reports included in the nutrition-related systematic reviews are identified through searches of online electronic databases, supplemented by hand reviews of journals, and by examination of bibliographies, as necessary, following a search plan developed by a work group with expertise in the specific nutrition topic being evaluated. Identified research reports are checked to verify that each one met the pre-established eligibility criteria. Trained analysts then reviewed each research report and abstracted pertinent information into an online worksheet. A QCC, either for primary research [Figures 2 and 3 **]** or reviews of research [Figure 4], was used to appraise the research report and determine an overall research quality rating of positive (higher quality), neutral, or negative (lower quality). The overall quality ratings indicated the quality of the research design and implementation of the research as shown in the research report, but did not indicate the direction or nature of the findings. Ratings to the sub-questions were made according to the research design and used to formulate the rating for the domain question. Worksheets and QCC responses were reviewed by a lead analyst, and following approval by the expert workgroup, were approved for inclusion in the systematic review. A full description of the appraisal process is available on the Evidence Analysis Library website(www.adaevidencelibrary.com) and in excerpts from the *Evidence Analysis Manual* which can be found in the supplemental material for this article [Supporting Information S1]. As of February 2009, over 2,600 abstracted research reports in 23 nutrition topics of priority interest to the field (e.g., management of hypertension, adult and pediatric obesity, diabetes, disorders of lipid metabolism, non-nutritive sweeteners, and nutrition counseling) were included in the online evidence analysis library [Figure 5]. Additional descriptions of the nutrition-related topics included in the sample are found in the supplemental materials for this article [Supporting Information S2].

The availability of an existing evidence analysis database that included a large number of nutrition-related research report quality appraisals provided a unique opportunity to investigate the relationships between funding source and research report quality. The aim of this cross-sectional study was to determine whether funding source of nutrition and dietetics practice-related research, particularly industry-funded research, was associated with differences in research report quality, and further, if these associations varied across different types of research design. In this study, “quality” refers to a set of expectations for the design, implementation, and reporting of research that are believed to reduce the risk of bias and support the validity of findings. Quality is assessed based on information available in published research reports utilizing checklists that incorporate widely accepted domains of rigorous scientific investigation and research synthesis. Thus, the term *research report quality* is used.

## Materials and Methods

### Sample

All research report appraisal forms already in the Evidence Analysis Library as of February 2009 were screened for complete and valid answers to the ten-item QCC. Research report appraisals were already included in the library database through the process described in the introduction and more completely described in the supplementary material and on the website. Out of a total of 2,632 entries, 93 were excluded resulting in a sample size of 2,539 research reports with overall quality ratings and completed QCCs.

### Variables

This study used the following variables: overall quality rating (positive, neutral, or negative), individual QCC domain responses (yes, no, unclear or not applicable), type of research (intervention, observation, and review), and funding source (government, industry, multiple, university/hospital, non-profit, and not reported).

### Data Collection

All data variables, with one exception, already existed in the database from previously completed systematic reviews. The funding source for each research report was the only missing data needed to complete this study. A consolidated report was created from the library database that extracted citations, research design, overall quality rating, and responses to individual QCC domain ratings. For this study, the published research reports included in the sample were examined to identify funding sources from the acknowledgements, report text, or author affiliations. The funders' names were recorded and, if necessary, the type of organization was determined after reviewing the respective organization's website. Research assistants, with no knowledge of the previously assigned quality ratings, classified the research reports into six funding source categories: government, industry, multiple, university/hospital, non-profit, and not reported. If funding from more than one funding source category was recorded, the research report was placed in the multiple funders category; however, if the research reported two or more funders from the same category (e.g., two non-profits), the report was classified in the same category.

The research design recorded in the abstract worksheet during the evidence analysis process was used to assign each research report into one of three categories for research-type: intervention, observation, and review. The intervention category included randomized controlled trials, non-randomized trials, and non-controlled trials. The observation category included cross-sectional, cohort, case-control, and other observational research. The review category included narrative reviews, systematic reviews, and meta-analyses.

The QCC data extracted from the library database were combined with the newly created data for assigned funding source and research-type category created for data analysis.

### Analyses

Given the conflicting research on the quality of research reports funded by industry sources, we hypothesized that after research design was taken into account, there would be no difference in overall quality rating between those research reports funded by government sources and those funded by industry sources. Secondarily, we sought to quantify the effect size of the funding source/research report quality relationship in order to determine whether detected relationships were meaningful, because findings of statistical significance do not equate to practically meaningful differences (referred to as “clinical significance” in clinical settings), particularly in a large data set [43].

An a priori power calculation based on effect sizes gleaned from a similar study (at α = 0.05 and a power of 0.8) indicated that a sample size of 241 industry-funded research reports was needed for a chi-square analysis [44]. However, because of differences in methodologies of the previous study and our current study, this value was used as an approximation. A post hoc test of power was calculated to examine the difference in proportion in quality ratings between government and industry funding sources and revealed an achieved power of 0.99.

Descriptive statistics are reported using frequency distributions (n, %). In addition, to determine whether research design should be included in the model as a confounder, chi-square tests were used to examine possible associations between the research-type category and the funding source and the research report quality, respectively. A comparison of the funding source and the type of research of the 93 excluded research reports verified that they were not significantly different from the total sample (funding source: χ^2^ = 3.53, *P* = 0.474; research-type category: χ^2^ = 4.22, *P* = 0.121).

We used hierarchical multinomial logistic regression to model and test predictors of research report quality rating, with both the funding source and research-type category serving as independent variables [45]. The term *government* was set as the reference category for funding source, and *intervention* became the reference category for the research type. These reference categories were selected because government funding is typically viewed as more credible, and interventions that include randomized controlled trials (RCT) are considered the gold standard of research design. Changes in -2 log likelihood were used to determine whether each independent variable contributed significantly to the model, and differences between categories of the independent variables were tested using the Wald statistic [46]. The effect size of individual categories is reported using odds ratios (ORs). The Nagelkerke pseudo *R* ^2^ was used as a metric of effect size of the overall model. In order to obtain further insights, three modified versions of the above analysis were carried out. First, the model was also run with other reference standards (e.g., industry and review, and government and observation), and these analyses did not provide additional insights. Second, the same analysis was repeated except with review design studies dropped from the analysis. Finally, RCT design studies were separated from other types of intervention trial designs and we tested for an association between study reporting quality and funder type within each different type of study design. Because of small cell size, a chi-square test of independence rather than a logistic regression was used for this final analysis.

An a priori alpha value was set at *P*≤0.05. SPSS software version 17.0 (SPSS Corporation, Chicago, Illinois) was used for all analyses.

A series of secondary analyses were done to describe ratings for individual quality items for reports of studies with intervention and observational study designs and to examine the association of individual quality criteria with funding source; and to investigate the possible impact of review articles on the research findings conclusions. Chi-square tests were used to confirm expectations that the four questions required for positive quality rating were associated with quality ratings. Because of the QCC instructions [Figure 2, 3 and 4], we anticipated that questions 2, 3, 6 and 7 would be significant predictors of positive quality.

Multinomial logistic regression analyses were used to determine the relative importance of each question's contribution to overall quality rating. Positive quality rating was set as the reference category for the first model (neutral or negative quality compared to positive) and neutral quality was set as the reference category for the second analysis in order to determine which questions predicted negative compared to neutral quality. “No” and “unclear” responses were collapsed into “no” for this analysis.

Because of a complete separation of data for questions 2, 3, 6 and 7 (since these questions were required for an overall positive rating), we repeated the first analysis (positive versus neutral and negative) with a reduced model (omitting these four questions). Odds ratios and 95% confidence intervals around the ORs were calculated to determine which QCC questions best predicted quality rating among intervention and observational studies.

## Results

### Overall Quality Ratings

Descriptive statistics showing the research-type and funding sources are shown in Table 1. The most common research-type categories in the sample were intervention (51.6%) and observation (39%), with the review type comprising only 9.4% of the research reports. The multiple funders category (n = 762, 30%) had the largest number of research reports, followed by the university/hospital category (n = 665, 26.2%). The multiple funders category included 353 research reports (45%) that had industry as one of the multiple funders; and 183 of those research reports included a combination of government and industry funding. The industry funder category included food manufacturing companies (n = 100), pharmaceutical companies (n = 81), commodity groups, (n = 13), and other funders (n = 17). The research-type category was significantly associated with the funding source (χ^2^ = 126.95, *P*≤0.001). Research reports funded by government and university/hospital funding sources were more evenly divided between the categories of intervention and observation, whereas a higher proportion of industry-funded research reports was found in the intervention category. Reviews were more commonly funded by non-profits, followed by university/hospital sources.

**Table 1:** Nutrition-related research reports by funding source and type of research.^1^

Type of Research								
	Intervention^2^		Observation^3^		Review^4^		Total	
Funding Source	n	(%)	n	(%)	n	(%)	n	(%)
Government	249	(48.9)	229	(45.0)	31	(6.1)	509	(20.0)
Industry	147	(73.9)	35	(17.6)	17	(8.5)	199	(7.9)
Multiple funders	429	(56.3)	288	(37.8)	45	(5.9)	762	(30.0)
University/hospital	313	(47.4)	268	(40.3)	84	(12.6)	665	(26.2)
Non-profit	77	(46.1)	52	(31.1)	38	(22.8)	167	(6.6)
Funder not reported	95	(40.1)	119	(50.2)	23	(9.8)	237	(9.3)
Total	1310	(51.6)	991	(39.0)	238	(9.4)	2539	(100.0)

1 Funding source and type of research are significantly associated (χ^2^ = 126.95, *P*<0.001).

2 Intervention research includes research designs such as randomized (individual and group), non-randomized, and non-controlled trials.

3 Observation research includes cross-sectional, cohort, case control, time series, trend, and non-comparative studies.

4 Review research includes narrative and systematic reviews and meta-analyses.

The overall distribution of quality ratings for research reports was 43.3% positive, 50.1% neutral, and 6.6% negative. As shown in Table 2, the proportion of quality ratings differed significantly by research type (χ^2^ = 89.64, *P*<0.001).

**Table 2:** Nutrition-related research reports by type of research and quality rating.^1^

Quality Rating								
	Positive^2^		Neutral^3^		Negative^4^		Total	
Type of Research	N	(%)	N	(%)	n	(%)	n	(%)
Intervention	575	(43.9)	663	(50.6)	72	(5.5)	1310	(51.6)
Observation	454	(45.8)	490	(49.5)	47	(4.7)	991	(39.0)
Review	71	(29.8)	118	(49.6)	49	(20.6)	238	(9.4)
Total	1100	(43.3)	1271	(50.1)	168	(6.6)	2539	(100.0)

1 Quality rating and type of research are significantly associated (χ^2^ = 89.64, *P*<0.001).

2 A positive quality rating requires that four critical quality criteria (selection of subjects free of bias, study groups comparable, intervention or procedure and intervening factors described, and outcomes clearly defined and measured using valid and reliable methods) are met and at least one additional checklist item is met.

3 A neutral quality rating is assigned when responses to the four critical quality criteria do not indicate that the research report is exceptionally strong.

4 A negative quality rating is assigned when six or more of the 10 checklist items are not met.

Table 3 shows the results of the multinomial logistic regression to test the hypothesis that funding source predicts research report quality when controlling for the research type category. Although the model combining both funding source and research type met the criteria for statistical significance (χ^2^ = 118.99, *P*<0.001), the effect size is very small (pseudo *R* ^2^ = 0.055). Based on -2 log-likelihood statistics, both independent variables, funding source and research design type, contribute significantly to the model (χ^2^ = 52.89, *P*<0.001; and χ^2^ = 62.81, *P*<0.001, respectively). Although both independent variables were significant predictors of research report quality, the predictive accuracy of the model was, overall, little better than chance. Based on a cross-classification of predicted quality by actual quality, the model only correctly classified 50.9% of research reports. The model predicting research report quality from funding source and research type correctly classified none of the negative quality research reports, 70% of the neutral quality research reports, and only 36.6% of the positive quality research reports.

**Table 3:** Predictors of nutrition-related research manuscripts receiving neutral and negative quality ratings versus positive ratings compared with government-funded intervention research.^1^

	Likelihood of Quality Rating Versus Positive Quality			
	Neutral		Negative	
Factor	OR	95% CI of OR	OR	95% CI of OR
Funder				
Government (reference category)	1.00	—	1.00	—
Industry	1.38	0.98–1.95^2^	1.90	0.95–3.81
Multiple funders	1.10	0.87–1.38	0.98	0.56–1.71
University/Hospital	1.54^3^	1.21–1.96	1.62	0.95–2.76
Non-profit	1.17	0.81–1.68	1.14	0.53–2.45
Not reported	1.85^3^	1.32–2.59	4.97^3^	2.76–8.95
Type of research				
Intervention (reference category)	1.00	—	1.00	—
Observation	0.92	0.78–1.10	0.77	0.52–1.15
Review	1.38^4^	1.00–1.90	5.26^3^	3.34–8.28

Abbreviations: CI, confidence interval; OR, odds ratio.

1 Statistical significance of the model combining funding and type of research to predict quality ratings (χ^2^ = 118.99, *P*<0.001, pseudo *R* ^2^ = 0.055).

2 Confidence intervals containing the value of 1.0 do *not* indicate a statistically significant difference between the response and reference category.^37^

3 *P*<0.001, based on the Wald statistic.

4 *P*<0.05, based on the Wald statistic.

Only two funding sources (university/hospital and not reported) were significantly more likely to receive an overall neutral quality rating than research reports with government-only funding (OR = 1.54, *P*<0.001; and OR = 1.85, *P*<0.001, respectively). After controlling for research type (*P* = 0.069), results indicated that research reports funded by industry sources were no more likely to receive a neutral quality rating than those funded by government sources. With respect to a negative quality rating, only research reports in which the funding source was not reported were more likely than government-funded research reports to receive a negative quality rating (OR = 4.97, *P*<0.001). Except for very slight differences in the OR values, results were identical for models that included only intervention and observational research and excluded review design studies. Thus, the relationship between funding source and research report quality was not a result of including review design studies in the model.

Review research type reports were more likely to receive both neutral (*P*<0.05) and negative (*P*<0.001) quality ratings than intervention research type reports. Observation research-type reports were no more likely to receive either a neutral or a negative quality rating than intervention research type reports (*P* = 0.367 and *P* = 0.200, respectively).

In the final analysis, RCT design studies were separated from other types of intervention trials (resulting in four research design types: review, observational, RCT, and other intervention types). There was no statistically significant association between funder type and research reporting quality within either review (χ^2^ = 17.78, *P* = 0.059) or observational (χ^2^ = 17.69, *P* = 0.060) study types. There was a significant association between funder type and reporting quality within RCT designs (χ^2^ = 49.35, *P*<0.001). A valid chi-square test was not possible within other intervention design studies because more than 20% of cells contained an expected value of <5. Within the RCT design, studies funded by multiple funders were significantly less likely to be negative quality while studies where funding was not reported were significantly more likely to receive a negative quality rating.

### Individual QCC Domain Responses in Primary Research Reports

In our sample of primary research reports, all of the QCC domain questions received a “yes” response in at least 50% of the articles, with only the following two questions receiving this answer in less than 70% of articles: whether subject selection was free from bias (Q2, 66.52%) and whether blinding was used (Q5, 50.31%). These three QCC domain questions received responses of “unclear” in at least 10% of the articles: whether subject selection was free from bias (Q2, 23.33%), whether study groups were comparable (Q3, 11.79%), and whether blinding was used (Q5, 11.84%).

Table 4 shows the domains from the primary research QCC, which were more likely to have a “no” or “unclear” response by funder category reflecting a weakness, or less likely to receive either “unclear” or “no” reflecting a strength in the primary research reports at the *P* <0.05 significance level. The funding categories with strengths in research report quality are: multiple funders (4 strengths), government (3 strengths), and non-profit (2 strengths). The funding categories with weakness are no funding reported (7 weaknesses), university/hospital (5 weaknesses), industry (1 weakness), and government (1 weakness). Only two of the criteria identified as strengths were repeated in more than one funding category. Both multiple funders and government had significantly fewer “no” or “unclear” responses on Question 9 (conclusion support) and Question 10 (likelihood of bias due to funding). University/hospital and not reported funding categories both showed weaknesses in Question 3 (group comparability), Question 6 (intervention process), and Question 8 (analysis). Government and not reported funding categories both received a higher proportion of responses as “no” or “unclear” for Question 5 (blinding). University/hospital and industry both reported a higher proportion of responses as “no” or “unclear” to Question 10 (likelihood of bias due to funding). In addition to identifying these responses by funding category, an additional analysis was completed to determine if Question 10.2 (likelihood of conflict of interest) was sufficient to predict the overall quality rating. When evaluating all overall quality ratings together, the rating for Question 10 (likelihood of bias due to funding) was not a statistically significant predictor (*P* > 0.05) of either an overall neutral or negative quality rating compared to a positive overall quality rating.

**Table 4:** Primary research report areas of strengths and weaknesses based on Quality Criteria Checklist domain responses.^1^

	Domain	Multiple Funders	Government	Industry	University/hospital	Non-profit	No Funding Reported
Q1	Research Question						
Q2	Subject Selection	Strength^2^					Weakness
Q3	Comparable Group	Strength			Weakness		Strength
Q4	Withdrawals					Strength	Weakness
Q5	Blinding		Weakness^3^			Strength	Weakness
Q6	Intervention/ Exposure		Strength		Weakness		Weakness
Q7	Outcome				Weakness		Weakness
Q8	Analysis						Weakness
Q9	Conclusion Support	Strength	Strength		Weakness		
Q10	Likelihood of Bias Due to Funding	Strength	Strength	Weakness	Weakness		

1. Each of the 10 Quality Criteria Checklist domain responses could be “yes”, “no” or “unclear.”

2. Strength indentified if *P* values based *Z* distribution of chi-square standardized residuals is <0.05 for: (a) greater than expected “yes” responses, (b) lower “no” responses, (c) lower “unclear” responses, (d) any combination of the above.

3. Weakness identified if *P* values based on *Z* distribution of chi-square standardized residuals is <0.05 for (a) lower than expected “yes” responses, (b) higher “no” responses, (c) higher “unclear” responses alone, or (d) lower “unclear” responses in combination for (b).

4. Cells with no value indicate that observed frequencies did not deviate from what would be expected if variables were independent.

## Discussion

In this study, funding source was found to provide only minimal information about the quality of the research report. While the statistical results support the hypothesis that an association exists in nutrition-related research between the funding source and overall quality rating for research reports after controlling for the type of research design; our research indicated that the effect size of the relationship was very small. The model for predicting research report quality from the funding source and research type allows us to predict only marginally better than chance (50.9%).

The more specific hypothesis that nutrition-related research reports that received industry funding were of lower quality than those funded by government sources was rejected. After controlling for the research type category, nutrition-related research reports acknowledging industry funding were no more likely to be of neutral or negative quality than those that received government funding. Stated in general terms, this means that in nutrition-related research, research reports that reported industry funding cannot be assumed to be of lower quality than those funded by government sources [24]. It is also worth noting that although 40.5% of research reports in the multiple funders category had industry funding, this category was also not significantly different and more closely approximated the quality ratings of the government funding category.

In our sample, industry funded more intervention research type reports than observation studies or reviews (approximately 74%, compared with 40%–56% for other funders). This funding is not surprising because the burden of proof of the effectiveness of nutrients and food products to support health claims and marketing materials falls on industry [47]. Because review research type reports were disproportionately rated as being of negative quality, the higher proportion of reports in this research type category affected the distribution of the quality of research reports funded by non-profit and university/hospital sources.

The appraisal of the quality of research reports is integral to the process when systematic reviews are conducted for the purpose of supporting the development of clinical practice guidelines, serving as the basis for public policy, or identifying research needs [27], [47]. The peer-review process is intended to bring a high level of scrutiny to the appraisal process; however, even that level of expert review is not always sufficient to identify concerns. It is widely acknowledged that there are few if any perfect research studies and the limitations need to be carefully identified, discussed, and implications of limitations incorporated into the interpretation of the findings. It is therefore critical that quality appraisal of research reports is included in any synthesis of a body of research to avoid the possibility of magnifying any bias contained within that research. The intent is to rely more heavily on the higher-quality research reports that provide the best insight into the true nature of a phenomenon when developing systematic reviews. This would be equally true for primary research as well as review research (synthesized into systematic reviews, meta-analysis, or narrative reviews). The goal is to rely more heavily on research reviews that have the lowest risk of bias. Evaluation of whether the results are likely to be biased by a researcher's funding source is part of the overall quality appraisal process; however, the process is intended to reflect the quality of all aspects of the research report.

The examination of the QCC domains in primary research that were strengths and weaknesses in the published research reports in the library database by funding source highlights the relative similarities and differences between research reports by funding category. The response for Question 10 (likelihood of bias due to funding) is a strength of multiple funders (of which 45% include industry funding) and government-funded research reports, while this was categorized as a weakness in both industry and university/hospital funding. While this individual domain (likelihood of bias due to funding) is identified as a weakness in industry-funded research reports there were no other domains identified as either strengths or weaknesses for this type of funder. The large number of weaknesses identified in research reports from the not reported funding (7 weaknesses) and of university/hospital funding (5 weaknesses) categories identifies significant opportunities for improvement.

The present study supports the concept that although funding and research type are associated with the overall quality rating, simply knowing the funding source is insufficient to determine the quality of the research or its reporting.

### Limitations and Strengths

Although our findings indicate that industry funding is not associated with lower research report quality, our results do not rule out other avenues for bias. Issues other than the rigor of the research and reporting such as selection of topics to be researched, specific research hypothesis tested, or selective reporting of research report results in subsequent research papers, and publication bias by journals continue to be factors in nutrition-related and other research and warrant further attention [48], [49]. These findings also reflect only the content that was included in the published research article. It is not known whether narrative review research reports in the review category had a systematic method of selecting the summarized research if that information was not included in the published research reports.

The small number of QCCs for reviews precluded analysis at the QCC domain level to identify opportunities for improvement in research reviews.

Although the large research sample used in this investigation included a broad range of topics, it is limited to nutrition-related research that is relevant to dietetics practice. The generalizability of these results to other nutrition topics and research is unknown.

Our research did not explore the accuracy of the financial disclosures. Explicit identification of the funding source was lacking for some research reports classified in the university/hospital category, in which authors acknowledged support from their institution but it was unclear whether the support went beyond employment. In addition, our research did not determine the impact of the lack of sufficient funding. Only the not reported and the university/hospital funding categories had quality ratings that were significantly lower than government-funded research. Other data indicate that there may be systematic underreporting of industry financial support [50]. Some bioethics researchers question whether the criteria for financial disclosure go far enough, and suggest that these criteria be even more explicit and disclose ultimate funding sources for organizations supported by industry [17], [51], [52]. Although we reviewed websites to characterize funding organizations, it is possible that not all instances of industry funding were identified.

This study has two important strengths: the breadth of the nutrition-related topics covered, and a sample size that allows for us to control for known confounders (i.e., type of research design) and conduct a more refined differentiation among six different types of funder categories.

### Implications and Future Research

Review research reports had the largest opportunity for improvement in quality, regardless of the funding source. Because review research reports were consistently lower in quality and were frequently funded by nonprofits and industry, this may be an opportunity for these organizations and journal editorial staff to specify a preference for systematic reviews and meta-analyses, rather than more traditional narrative reviews.

The domains reflected as weaknesses shared by more than one funding category may represent the largest opportunity for improvement in nutrition-related research. Researchers may want to place additional emphasis on establishing and documenting group comparability or use of randomization, describing the intervention process, selecting and documenting appropriate outcomes measures, and likelihood of bias due to funding.

The present study lends support for the legitimate role of industry-funded research by dispelling a common concern that industry-funded research may be biased due to less rigorous research standards. Sensationalist headlines citing the direction of findings without also evaluating the scientific merit of the research are not helpful, and could lead to distrust of research in general without actually improving the research enterprise. Furthermore, if journal policies limit publication of industry-funded research, as some have suggested, the research is not readily available to inform the rest of the research enterprise or the public, which could limit the transparency of regulatory decisions [52]. Significant increases in federal funding would be required if industry-funded research were not considered as credible, because the greater burden for funding would be transferred to the government. Industry may want to consider increasing collaboratively funded research since the multiple funder category (of which 45% already included industry funding) had the greatest number of strengths and no weakness based on the individual QCC domain responses [Table 4]. However, it is critical to be vigilant so that all published research, regardless of the funding source, is of the highest possible quality.

Our research has implications for the media and consumers if the expectation or hope is that they judge the research on its merit, along with reporting the funding source [53]. Greater levels of commitment and research expertise are needed to evaluate the methods, statistics, and findings in a published research report to determine if there is a likelihood of bias in the research. The increased use of research summaries on websites makes it even more challenging for consumers to ascertain whether a research report has been peer reviewed and who funded the research and/or website. In general, the research literacy of consumers and the media would need significant enhancement if this were to be the desired end state.

Additional studies in the following areas may be beneficial in the future:

- evaluation of the accuracy of financial disclosures by researchers to determine usefulness in identifying the type of research funding, especially when their employer is the funding source acknowledged;
- assessment of the impact of recently published standards for publishing manuscripts in nutrition-related research because other disciplines report mixed results [24], [54]–[58];
- evaluation of the consistency in quality ratings among research quality appraisal tools;
- replication of the present study to determine if our findings are consistent in other systematic review databases (e.g., Cochrane or AHRQ databases); and
- determination of whether specific criteria in the checklists other than the Primary Research QCC used in this study confirm the domain areas most in need of improvements in nutrition-related research reports, and whether they are related to the direction of research findings (e.g., supportive of funders' interests).

### Conclusion

Overall quality of research reports cannot be accurately predicted from the funding source after controlling for research design. Our results showed that there was no evidence of bias reflected by lower research report quality ratings that could be attributed to industry funding sources in food and nutrition research included in the systematic reviews published in the online ADA Evidence Analysis Library. The lowest overall quality ratings and the most individual QCC domain weaknesses were assigned to research reports that did not acknowledge any funding sources, followed by those that acknowledged university/hospital funding. Continued vigilance to evaluate the quality of all research regardless of funding source and to further understand other factors that affect research quality are warranted. There may be benefits of external funding on quality of research regardless of the funding source, in particular with projects that receive multiple funding sources.

## Supporting Information

Supporting Information S1 **Evidence Analysis Manual, Chapter 3.**
(PDF)

Supporting Information S2 **Summary of Topics and Keywords from the Evidence Analysis Library database on February 2009 (expansion of** **Figure 5** **).**
(DOC)
