# Supplementary material for: Funding Source and Research Report Quality in Nutrition Practice-Related Research
Source: PLoS One. 2011 Dec 6;6(12):e28437. doi: 10.1371/journal.pone.0028437 (PMC3232225; doi:10.1371/journal.pone.0028437)
Supplement: Supporting Information S1 — Evidence Analysis Manual, Chapter 3. (PDF) [file pone.0028437.s001.pdf]

Evidence Analysis Manual

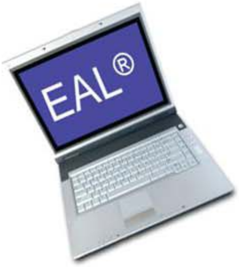

Steps in the ADA Evidence Analysis Process

Research and Strategic Business Development
January 2010

American Dietetic Association

ISBN: 978-0-88091-429-1
CatN: 4297

EVIDENCE ANALYSIS MANUAL: STEPS IN THE ADA EVIDENCE ANALYSIS PROCESS

Chapter 3 and related Appendices Excerpted

ISBN: 978-0-88091-429-1

Research and Strategic Business Development
American Dietetic Association
120 South Riverside Plaza
Suite 2000
Chicago, IL 60606-6995
800/877-1600
www.eatright.org
www.adaevidencelibrary.com

Updated January 2010

Copyright 2009, American Dietetic Association. All rights reserved. No part of this publication may be reproduced, stored in a retrieval system, or transmitted in any form or by any means without the prior written consent of the publisher. Printed in the United States of America.

The views expressed in this publication do not necessarily reflect policies and/or official positions of the American Dietetic Association. Mention of product names in this publication does not constitute endorsement by the authors or the American Dietetic Association. The American Dietetic Association disclaims responsibility for the application of the information contained herein.

Table of Contents

PREFACE............ERROR! BOOKMARK NOT DEFINED.

CHAPTER 1: FORMULATE EVIDENCE ANALYSIS QUESTION ....ERROR! BOOKMARK NOT DEFINED.

CHAPTER 2: GATHER AND CLASSIFY EVIDENCE……………………………………….…….17

CHAPTER 3: CRITICALLY APPRAISE EACH ARTICLE…………………………………………………..31

CHAPTER 4: SUMMARIZE EVIDENCE…………………50

CHAPTER 5: WRITE AND GRADE THE CONCLUSION STATEMENT……………………………………….…..….59

THE FINAL STEP.ERROR! BOOKMARK NOT DEFINED.

APPENDIX 1: QUESTION FORMULATION TEMPLATE…………………………………………………...66

APPENDIX 2: THE PICO FORMATERROR! BOOKMARK NOT DEFINED.

APPENDIX 3: SEARCH PLAN & RESULTS TABLE ....ERROR! BOOKMARK NOT DEFINED.

APPENDIX 4: CLASSES OF EVIDENCE REPORTS ....ERROR! BOOKMARK NOT DEFINED.

APPENDIX 5: ALGORITHM FOR CLASSIFYING RESEARCH DESIGNERROR! BOOKMARK NOT DEFINED.

APPENDIX 6: GLOSSARY OF TERMS RELATED TO RESEARCH DESIGNERROR! BOOKMARK NOT DEFINED.

APPENDIX 7: EVIDENCE ABSTRACT WORKSHEET TEMPLATE 23

APPENDIX 8: QUALITY CRITERIA CHECKLIST: PRIMARY RESEARCH.. 25

APPENDIX 9: QUALITY CRITERIA CHECKLIST: REVIEW ARTICLE.. 28

APPENDIX 10: IMPORTANT CONSIDERATIONS FROM CHECKLIST BY STUDY DESIGN................................ 29

APPENDIX 11: TALLY SHEET OF QUALITY CRITERIA RATINGS 30

APPENDIX12: SAMPLE TALLY SHEET FROM THE EAL® 31

APPENDIX 13: OVERVIEW TABLE TEMPLATE ERROR! BOOKMARK NOT DEFINED.

APPENDIX 14: EXAMPLE OF AN ADA OVERVIEW TABLE FROM THE EAL®ERROR! BOOKMARK NOT DEFINED.

APPENDIX 15: CONCLUSION STATEMENT AND GRADE .... ERROR! BOOKMARK NOT DEFINED.

APPENDIX 16: GRADE DEFINITIONS: STRENGTH OF THE EVIDENCE FOR A CONCLUSION/RECOMMENDATIONERROR! BOOKMARK NOT DEFINED.

APPENDIX 17: CONCLUSION GRADING TABLEERROR! BOOKMARK NOT DEFINED.

Chapter

3

Step 3: Critically Appraise Each Article

Instructions for Abstracting an article onto the Evidence Worksheet

An analyst is responsible for critically reviewing each research article and abstracting key information on to the *Evidence Worksheet*. The abstracted information on the *Evidence Worksheet* is used later by the expert panel to write the conclusion statement (answer to the question) and grade the strength of the evidence. Information from all of the worksheets is included in the Evidence Overview Table that supports the conclusion statement.

There are several documents that will help you to complete the *Evidence Worksheet*:

- "Tips": (primary and review article) tips for how to complete the worksheets are found in Table 3.0 and Table 3.1. Additional suggestions are in Table 3.2.
- *Quality Criteria Checklists*: checklists of questions to help you determine the relevance and validity of primary and review articles—found in Table 3.3 and Table 3.4 and Appendix 8 and Appendix 9 in the Appendices section
- Study Design Table: a table that indicates which questions from the quality criteria checklists are most relevant for different study designs—found in table 3.5 and Appendix 10 in the Appendices section

This chapter will describe how to use these tools to accurately complete the *Evidence Worksheet* for each included article on the *Search Plan & Results*.

Action 7: Abstract Key Information from the Research Article into the *Evidence Worksheet*

Before you attempt to abstract details about the study into the worksheet, you will need to read carefully the article. While abstracting the article, pay close attention to the study design and execution elements that affect the scientific validity of the work.

WHY IS THE WORKSHEET SO IMPORTANT?

Purpose of the Worksheet

The worksheet provides an organized way to:

- Abstract key information for future reference.
- Identify study details that allow determination of study quality.
- Summarize major findings including the magnitude of effect and the statistical significance and/or confidence interval.
- Record the author's conclusion.
- Note reviewer's comments about the study limitations and applicability.
- Note the funding source.

Instructions for Filling out the Evidence Analysis Worksheets

Below is a brief description of how to begin taking key information from the research article and transferring it into the worksheet. The process is somewhat different for primary research articles versus review articles.

Primary Research

Read the article to determine the purpose and population studied. Look for details about study design, criteria for study eligibility, the practice studied, study protocol, and the variables measured in the Method section. Find results in the text and tables of the Results section. See how the author interprets the findings and describes any limitations of the study in the Discussion section. Usually the author closes the article with a concise conclusion of the study. Transfer relevant information onto the *Evidence Worksheet*. (Refer to Table 3.0 for tips on what to abstract from Primary Research.

During the abstracting, use the *Quality Criteria Checklist* for primary research to assess the quality constructs and domains identified in the AHRQ report on *Systems to Rate the Strength of Scientific Evidence (2002)*[3].

[3]"Systems to Rate the Strength of Scientific Evidence". Agency for Healthcare Research and Quality (AHRQ) March 2002

Secondary Research or Reviews

Most review articles are organized in the same way as primary research reports. The key difference is that in a review article, the published research studies are the "subjects" of the study. Look in the report to find the purpose, population studied, and context for the review. Details about the search plan, criteria for study eligibility, the interventions, procedure and/or factors and outcomes of interest, methods for assessing quality of articles and abstracting data should be found in the method section. These details are described in a systematic review or meta-analysis, but generally have been less structured in narrative reviews. Find results in the text and tables of the results section. Note how the author interprets the findings and describes any limitations of the study in the discussion section. An author usually closes the article with a concise conclusion of the study. Transfer relevant information onto the *Evidence Worksheet*. Refer to Table 3.1. for tips on what to abstract from Reviews.

During the abstracting process, use the *Quality Criteria Checklist* for review articles to assess the validity of the study.

Tips for Completing Primary Research and Review Article *Evidence Worksheets*

Below, we provide two *Evidence Worksheets* templates—one for primary research and the other for review articles—that include tips for filling in the appropriate information. You can find these in Table 3.0 and Table 3.1. A blank copy of the *Evidence Worksheet* is included in the Appendices.

Table 3.0 What to Abstract from Primary Research

**Citation:**	List the complete bibliographical citation
**Study Design:**	Name of the study design. Refer to algorithm (Figure 2.3)
**Class:**	(A, B, C, D) Based on classes of evidence reports (Table 2.3)
**Quality Rating:**	(+, Ø, -) Based on quality criteria checklist for primary research
**Research Purpose:**	Research question being investigated in study
**Inclusion Criteria:**	Requirement for study eligibility
**Exclusion Criteria:**	Items that disqualify an individual from participation in study.
**Description of Study Protocol:**	What happened in the study<br><br>Describe interventions, regimens, risk factors, or procedures studied; when outcomes were measured; how intervening factors were managed.
**Data Collection Summary:**	Outcome(s) and other indicators<br><br>Important variables and methods of measurement<br><br>Was blinding used?

**Description of Actual Data Sample:**	Relevant descriptors of sample and comparison of groups at baseline<br><br>Note loss of subjects (withdrawals, dropout, response rate, etc.)
**Summary of Results:**	Key Findings<br>Abstract results including quantitative data and statistics. Be specific. Often tables are created in this section.<br>(Include statistical significance – P values, confidence intervals, relative risk, odds ratios, likelihood ratio, number needed to treat, if available)
**Author Conclusion:**	As stated by the author in body of report
**Reviewer Comments:**	*Note strengths and limitations of the study. Identify concerns that affect study validity and generalizability (Always italicize)*
**Funding Source**	Who provided the funding for this study?

Table 3.1 What to Abstract from Review Article

**Citation:**	List the complete bibliographical citation
**Study Design:**	Type of review (systematic, narrative, meta-analysis)
**Class:**	(M, R, X) Based on classes of evidence reports
**Quality Rating:**	(+, Ø, -) Based on quality criteria checklist for reviews
**Research Purpose:**	Question being addressed in the research
**Inclusion Criteria:**	Criteria for article inclusion
**Exclusion Criteria:**	Why articles were excluded from review.
**Description of Study Protocol:**	Search procedures<br><br>Was study quality assessed?<br><br>Type of interventions and outcomes investigated, populations included
**Data Collection Summary:**	What type of information was abstracted from articles?<br><br>How was it combined?<br><br>What analytic methods were used, if any?
**Description of Actual Data Sample:**	<u># of articles included</u><br># of articles identified<br><br>Number and type of studies reviewed<br><br>Sample size of studies, and characteristics of the study participants
**Summary of Results:**	What are the main results of the review?<br>Be specific.<br><br>Abstract results including quantitative data and statistics, especially effect sizes<br>Tables that summarize results can be useful.
**Author Conclusion:**	As stated by the author in body of report
**Reviewer Comments:**	*Note strengths and limitations of the review. Identify concerns that affect the validity of the review. How generalizable are the findings? (Always italicize)*
**Funding Source**	Who provided the funding for this study?

Additional Suggestions from Experienced Analysts

This list was compiled from the experiences of evidence analysts and lead analysts who have been working on ADA projects for several years.

Table 3.2 Suggestions from an analyst

**Reviewer**	
**Citation**	Always fill in this part in correct format (American Medical Association Style) and italicize periodical title. Example: Author A, Author B. Title of article. *Title of Periodical* year; volume: first-last page.
**Study Design**	Be sure to get this right! Discuss with lead analyst if not sure (sometimes it is difficult to tell). To help you, refer to the following items in the Evidence Analysis Manual:<br>• Table 2.1 Hierarchy and Classification of Studies<br>• Figure 2.2 Algorithm for Classifying the Research Design of Primary Studies<br>• Appendix 5: Glossary of Terms Related to Research Design; and<br>• Appendix 8 Important Quality Considerations from Checklist by Study Design
**Class**	Do not forget to use the pull down menu and designate A, B, C, D, etc. Refer to the Evidence Analysis Manual, Appendix 3: Classes of Evidence Reports.
**PubMed ID:**	Find the citation on PubMed to get this number. Often the Lead Analyst includes this information when the article assignment is created.
**RATING:**<br><br>After completing the research design and implementation checklist, determine the rating.<br><br>**Plus/Positive (+)** *If most of the answers to the validity questions are "Yes" including criteria 2, 3, 6 and 7 and at least one additional "yes", the report should be designated with a plus symbol (+) on the Evidence Worksheet.*<br><br>**Minus/Negative (-)** *If most (six or more) of the answers to the validity questions are "no," the report should be designated with a minus (-) symbol on the Evidence Quality Worksheet.*<br><br>**Neutral (ø)** *If the answers to validity criteria questions 2, 3, 6 and 7 do not indicate that the study is exceptionally strong, the report should be designated with a neutral (ø) symbol on the Evidence Quality Worksheet.*<br><br>Ratings are based solely on the quality criteria checklist. If you have any comments about the rating, explain in the reviewer's comments section and/or bring it forward as an item for discussion by the expert workgroup.	
**Research purpose**	Statement of purpose or research question: usually one or two sentences.
**Inclusion criteria**	• Use bullet points<br>• Informed consent if mentioned
**Exclusion criteria:**	• Exclusion criteria 1

		• Exclusion criteria 2 , etc.<br>Note: sometimes exclusion criteria are the opposite of inclusion criteria. (e.g., include individuals over the age of 20 = exclude individuals 19 and younger)
**Description of Study Protocol**	**Recruitment or sampling**	• Examples:<br>o Recruited from clinics<br>o Consecutive admissions to ICU<br>o Random selection of charts with specified diagnosis
	**Blinding used**	List if the author mentions blinding of subjects, providers, and/or investigators/data collectors. If not mentioned we assume no blinding was used and the prompt – blinding used- *can be deleted* from worksheet template)
	**Description of study protocol:**	Give the highlights here including: type of comparisons or groups, method of assignment to groups (random, convenience, etc), number and timing of data collection points, and procedures for follow up of subjects. If there are treatment and control or comparison groups list them.
	**Intervention** (if applicable)	List the intervention, regimen, risk factors or procedures studied. Include specifics such as type, dose, duration or intensity. This is usually the independent variable. For example, counseling by dietitians and medication management to control blood glucose between 80 and 140 mg/dL. After the intervention information is added to the worksheet, *delete the prompt "if applicable."*
	**Statistical analysis**	• Name the statistical tests used<br>• Indicate if multivariate analyses were done to control or adjust for other variables<br>• Intent to treat analyses applies to any type of intervention study (pre-post, nonrandom trial and RTCs)<br>• Report the results of a power analysis if one was conducted. This is the probability that the test will reject a false null hypothesis (or Type 2 error). The author will say something like *n subjects were needed for 80% power.*
**Data Collection Summary:**	**Timing and method of measurements**	Examples:<br>• Hemoglobin A1c was tested at baseline and at quarterly clinic visits<br>• Subjects completed a validated food behavior checklist and were weighed at baseline, 6 and 12 months<br>• Pressure ulcer was staged according to xx criteria
	**Dependent variables** (outcomes)	Examples:<br>• Mortality (died while hospitalized)<br>• Change in hemoglobin A1c<br>• Percent of body weight lost
	**Independent variables** (intervention or procedure)	Examples:<br>• Blood glucose controlled between 80-140 mg/dL<br>• Method of nutrition counseling<br>• Specific formulation of enteral feeding product

		Note: some correlational, descriptive studies do not designate independent and dependent variables. In that case, just list key study variables.
	**Other Variables**	This is anything else the investigator is tracking to assure the validity of the study or to clarify the relationship between the independent and dependent variable. It could be a confounding or intervening factor, comorbidity, a concurrent treatment, or subject or setting characteristics. List things that are pertinent to the evidence analysis question and its application to dietetic practice<br>• Nutrition support protocol at 25 to 30 kcal per kg within 48 hours of admission.<br>• Disease severity<br>• Number of previous weight loss attempts
**Description of Actual Data Sample:**	**Initial n** (e.g. 731 (298 males, 433 females)	Be careful here. Sometimes the author will report how many were screened (e.g. N=850) as well as how many were actually entered into the study (n=731). Report the number who made it into the study on the worksheet. If you are in doubt, just list both. Be sure to specify the breakdown by gender (often described as percent male).
	**Final n** (attrition)	This accounts for dropouts. The purpose for including attrition is that loss of subjects leads to bias and weakens the validity of the study. It is especially problematic when the number lost or the reason for dropout is different between the intervention and control groups. Be careful here; sometimes a dependent variable is mortality. In that case, a subject death is not a drop out and the final n is the same as at the beginning (because the authors accounted for all the subjects) – include the % attrition. A good quality study has a dropout rate of <20%.
	**Age**	List the age range. There is almost always a Table with subject characteristics (demographic, anthropometric data, etc) compared across groups that will have P values. A difference is not significant unless P < 0.05.
	**Ethnicity**	List if information is available. If it is not described, then state "not described"
	**Other relevant setting characteristics**	Consider the question: may need to define type of setting (e.g. medical ICU, surgical ICU, mixed ICU, trauma unit), staffing pattern, reimbursement/coverage
	**Anthropometrics or other relevant subject characteristics**	Were groups same or different on important baseline measures like BMI?
	**Location**	Report the city, state and/or country. If it is a multi-center trial, specify which country (e.g, multi-center trial in United States)

**Summary of Results**	**Primary findings**	• It is good to make tables here, but you should not copy all the tables from the article.<br>• Abstract the findings most pertinent to the evidence analysis question. Include quantitative information about the magnitude of effect and include statistical

		significance.<br>• Put the results that pertain to the dependent variables. If you list something as a dependent variable, give information about the result.<br>• To be reported appropriately, we need to see significance levels like P values or odds ratios with confidence intervals<br>• If it was not significant, it may be helpful to summarize those points in a bulleted list.
	**Other findings**	Something you have not listed as a dependent variable but is useful information.
**Author conclusion**		<u>Summarize</u> what the author said. Sometimes this addresses the clinical significance of the findings.
**Reviewer comments**		*You do not have to put anything here. However, if there is a particular strength or limitation you feel is important, write it here. Anything you (the reviewer) write is to be italicized.*
**Funding Source**	**Government**<br>**Industry**<br>**University/Hospital**<br>**Not-for-Profit**<br>**Other**	Check all funding source categories that apply.<br>Enter the specific name of the funder in the text field box

Please enter all content in your worksheet according to the ADA style requirements listed below.

<u>**ADA Style**</u>:

Spacing:
- Use a single space after punctuation (not double-space)
- No comma before "and"
- No comma before "or"
- No spaces before and after =, < and > symbols (e.g., P>0.0001)
- Use an extra space after these symbols when the following number is negative (< -1).

Symbols
- Do not use a slash (/) to separate terms such as +/1; instead use ±,or the greater than or less than symbol which you can then format the font to underline (≤ or ≥)
- Write fractions as ¾ not 3/4.

Punctuation:
- Periods and commas belong inside end-quotes.
- All other punctuation goes outside end-quotes.
- Italicize *title of periodical.*
- Use subscripts and superscripts appropriately: O_2 not CO2; m^2 not m2
- P-Value expressed as capital P (P<0.001)
- Spell out integers zero through nine unless followed by decimal (one or 1.0)
- Decimals must be preceded by 0. (P<0.001) *not* (P<.001).
- Spell out percentile; *not* %ile
- Spell out units of time: minutes *not* min; seconds *not* sec, etc.

- Last bulleted item must end with punctuation
- Capitalize L for liter (e.g., dL and L) but *not* for milliter (*ml is correct*)
- Always use comma separators: 1,000,000.

Avoid the following frequent mistakes.

- Be sure to list units (*patients were followed up at 6,12 and 24)* Is that 6 weeks or 6 months?
- Define acronyms you are using on tables in the results sections. You can put a note under the table. For example not everyone would understand RYGBP. Spell it out somewhere as Roux-en-Y Gastric Bypass (RYGBP).
- Data always "are" (not "is") because data are plural. Datum is singular.
    - Nutrient data were obtained (**not** *data was* obtained)
- Watch your subject/verb agreement—this must be the most common grammatical error (examples)
    - "Patients received an internal medicine and psychiatry evaluation" (better to say *patients received internal medicine and psychiatry evaluations* or *patients received an internal medicine and a psychiatry evaluation*)
    - "If there are treatment groups and a control, list them" is correct even if the grammar checker says it should read "there is treatment groups and …" treatment is an adjective modifying groups. It would not be correct to say is (singular) groups (plural).

Action 8: Complete Quality Criteria Checklist and Determine a Rating

As the report is being examined, refer to the appropriate *Quality Criteria Checklist* to be reminded of the criteria for sound scientific research. The criteria are written in the form of yes/no questions to help the analyst examine the article for important details about the design of the study and its execution. Finally, the reviewer uses the *Checklist* to assign an overall rating to the study. Refer to Table 3.5 to see which questions are most relevant for each study design. A symbol indicating positive (+), neutral (∅), or negative (-) is selected from the dropdown tool on the *Evidence Worksheet* to assign the rating.

The task of critically appraising a research article is complex and requires time and concentration. At first, the process takes about 2 hours per article. Time is reduced as the analyst becomes more familiar with the research area and the use of the *Evidence Worksheet* and the *Checklist*. Using ADA's online tools facilitates the processes of abstracting articles and maintaining files.

Advantages of the *Quality Criteria Checklists*

The Quality Criteria Checklists were developed to assist the analyst in assessing the article's research design. Questions included in the criteria checklists address applicability to practice and scientific validity.

WHY USE THE *QUALITY CRITERIA CHECKLIST*?

The Quality Criteria checklists are used:

- To identify the concepts that are widely accepted as elements of sound scientific investigation
- To provide a tool to enable systematic, objective rating of primary research and review articles
- To support inter-rater agreement among reviewers/analysts.

Background of the Quality Criteria Checklist for Primary Research and Review Articles

The content of the *Quality Criteria Checklist* is based on the quality constructs and domains identified in the Agency for Healthcare Research and Quality (AHRQ) report on *Systems to Rate the Strength of Scientific Evidence* (2002).

Both checklists include four relevance questions that address applicability to practice and ten validity questions that address scientific soundness. The relevance questions and validity questions make up the criteria. These detailed checklists should guide the analysts and help them to recognize various threats that may undermine sound research and that could lead to invalid conclusions.

It is assumed that users of the *Quality Criteria Checklists* will have a graduate degree, an understanding of research and statistics, and will have completed training in ADA's Evidence Library Training Workshop.

When used by knowledgeable persons, the checklists should yield consistent results across raters. It is recommended that inter-rater agreement be examined and verified before embarking on a project.

Adapted from 2000 ©Joint Commission Resources: "A Practical Approach to Evidence Grading". Joint Commission Journal on Quality Improvement, Volume 26(12);707.Reprinted with permission.

Quality Criteria Checklist: Primary Research

The Quality Criteria Checklist: Primary Research includes ten validity questions based on the AHRQ domains for research studies. Sub-questions are listed under each validity question that identify important aspects of sound study design and execution relevant to each domain. Some sub-questions also identify how the domain applies in specific research designs. The Quality Criteria Checklist for Primary Research can be found in Table 3.3

Table 3.3. Quality Criteria Checklist: Primary Research

RELEVANCE QUESTIONS	
1. Would implementing the studied intervention or procedure (if found successful) result in improved outcomes for the patients/clients/population group? (NA for some Epi studies)	Yes No Unclear N/A
2. Did the authors study an outcome (dependent variable) or topic that the patients/clients/population group would care about?	Yes No Unclear N/A
3. Is the focus of the intervention or procedure (independent variable) or topic of study a common issue of concern to dietetics practice?	Yes No Unclear N/A
4. Is the intervention or procedure feasible? (NA for some epidemiological studies)	Yes No Unclear N/A
If the answers to all of the above relevance questions are "Yes," the report is eligible for designation with a plus (+) on the Evidence Quality Worksheet, depending on answers to the following validity questions.	
**VALIDITY QUESTIONS**	
**1. Was the research question clearly stated?**	Yes No Unclear N/A
1.1 Was the specific intervention(s) or procedure (independent variable(s)) identified?	
1.2 Was the outcome(s) (dependent variable(s)) clearly indicated?	
1.3 Were the target population and setting specified?	
**2. Was the selection of study subjects/patients free from bias?**	Yes No Unclear N/A
2.1 Were inclusion/exclusion criteria specified (e.g., risk, point in disease progression, diagnostic or prognosis criteria), and with sufficient detail and without omitting criteria critical to the study?	
2.2 Were criteria applied equally to all study groups?	
2.3 Were health, demographics, and other characteristics of subjects described?	
2.4 Were the subjects/patients a representative sample of the relevant population?	
**3. Were study groups comparable?**	Yes No Unclear N/A
3.1 Was the method of assigning subjects/patients to groups described and unbiased? (Method of randomization identified if RCT)	
3.2 Were distribution of disease status, prognostic factors, and other factors (e.g., demographics) similar across study groups at baseline?	
3.3 Were concurrent controls used? (Concurrent preferred over historical controls.)	
3.4 If cohort study or cross-sectional study, were groups comparable on important confounding factors and/or were preexisting differences accounted for by using appropriate adjustments in statistical analysis?	
3.5 If case control study, were potential confounding factors comparable for cases and controls? (If case series or trial with subjects serving as own control, this criterion is not applicable. Criterion may not be applicable in some cross-sectional studies.)	
3.6 If diagnostic test, was there an independent blind comparison with an appropriate reference standard (e.g., "gold standard")?	
**4. Was method of handling withdrawals described?**	Yes No Unclear N/A
4.1 Were follow up methods described and the same for all groups?	
4.2 Was the number, characteristics of withdrawals (i.e., dropouts, lost to follow up, attrition rate) and/or response rate (cross-sectional studies) described for each group? (Follow up goal for a strong study is 80%.)	
4.3 Were all enrolled subjects/patients (in the original sample) accounted for?	
4.4 Were reasons for withdrawals similar across groups?	

	4.5 If diagnostic test, was decision to perform reference test not dependent on results of test under study?	
**5.**	**Was <u>blinding</u> used to prevent introduction of bias?**<br>5.1 In intervention study, were subjects, clinicians/practitioners, and investigators blinded to treatment group, as appropriate?<br>5.2 Were data collectors blinded for outcomes assessment? (If outcome is measured using an objective test, such as a lab value, this criterion is assumed to be met.)<br>5.3 In cohort study or cross-sectional study, were measurements of outcomes and risk factors blinded?<br>5.4 In case control study, was case definition explicit and case ascertainment not influenced by exposure status?<br>5.5 In diagnostic study, were test results blinded to patient history and other test results?	Yes No Unclear N/A
**6.**	**Were <u>intervention</u>/therapeutic regimens/exposure factor or procedure and any comparison(s) described in detail? Were <u>intervening factors</u> described?**<br>6.1 In RCT or other intervention trial, were protocols described for all regimens studied?<br>6.2 n observational study, were interventions, study settings, and clinicians/provider described?<br>6.3 Was the intensity and duration of the intervention or exposure factor sufficient to produce a meaningful effect?<br>6.4 Was the amount of exposure and, if relevant, subject/patient compliance measured?<br>6.5 Were co-interventions (e.g., ancillary treatments, other therapies) described?<br>6.6 Were extra or unplanned treatments described?<br>6.7 Was the information for 6.4, 6.5, and 6.6 assessed the same way for all groups?<br>6.8 In diagnostic study, were details of test administration and replication sufficient?	Yes No Unclear N/A
**7.**	**Were <u>outcomes</u> clearly defined and the <u>measurements valid and reliable</u>?**<br>7.1 Were primary and secondary endpoints described and relevant to the question?<br>7.2 Were nutrition measures appropriate to question and outcomes of concern?<br>7.3 Was the period of follow-up long enough for important outcome(s) to occur?<br>7.4 Were the observations and measurements based on standard, valid, and reliable data collection instruments/tests/procedures?<br>7.5 Was the measurement of effect at an appropriate level of precision?<br>7.6 Were other factors accounted for (measured) that could affect outcomes?<br>7.7 Were the measurements conducted consistently across groups?	Yes No Unclear N/A
**8.**	**Was the <u>statistical analysis</u> appropriate for the study design and type of outcome indicators?**<br>8.1 Were statistical analyses adequately described the results reported appropriately?<br>8.2 Were correct statistical tests used and assumptions of test not violated?<br>8.3 Were statistics reported with levels of significance and/or confidence intervals?<br>8.4 Was "intent to treat" analysis of outcomes done (and as appropriate, was there an analysis of outcomes for those maximally exposed or a dose-response analysis)?<br>8.5 Were adequate adjustments made for effects of confounding factors that might have affected the outcomes (e.g., multivariate analyses)?<br>8.6 Was clinical significance as well as statistical significance reported?<br>8.7 If negative findings, was a power calculation reported to address type 2 error?	Yes No Unclear N/A
**9.**	**Are <u>conclusions supported by results</u> with biases and limitations taken into consideration?**<br>9.1 Is there a discussion of findings?<br>9.2 Are biases and study limitations identified and discussed?	Yes No Unclear N/A
**10.**	**Is bias due to study's <u>funding or sponsorship</u> unlikely?**<br>10.1 Were sources of funding and investigators' affiliations described?<br>10.2 Was there no apparent conflict of interest?	Yes No Unclear N/A
**MINUS/NEGATIVE (-)**<br>*If most (six or more) of the answers to the above validity questions are "No," the report should be designated with a minus (-) symbol on the Evidence Worksheet.*		

**NEUTRAL (∅)**<br>*If the answers to validity criteria questions 2, 3, 6, and 7 do not indicate that the study is exceptionally strong, the report should be designated with a neutral (∅) symbol on the Evidence Worksheet.*	
**PLUS/POSITIVE (+)**<br>*If most of the answers to the above validity questions are "Yes" (including criteria 2, 3, 6, 7 and at least one additional "Yes"), the report should be designated with a plus symbol (+) on the Evidence Worksheet.*	

Quality Criteria Checklist: Review Articles

The *Quality Criteria Checklist: Review Articles* has ten validity questions that incorporate the AHRQ domains for systematic reviews. These questions identify the systematic process for drawing valid inferences from a body of literature. The *Quality Criteria Checklist: Review Articles* can be found in Table 3.4.

Table 3.4 Quality Criteria Checklist: Review Articles

RELEVANCE QUESTIONS	
1. Will the answer if true, have a direct bearing on the health of patients?	Yes No Unclear N/A
2. Is the outcome or topic something that patients/clients/population groups would care about?	Yes No Unclear N/A
3. Is the problem addressed in the review one that is relevant to dietetics practice?	Yes No Unclear N/A
4. Will the information, if true, require a change in practice?	Yes No Unclear N/A
If the answers to all of the above relevance questions are "Yes," the report is eligible for designation with a plus (+) on the Evidence Quality Worksheet, depending on answers to the following validity questions.	
**VALIDITY QUESTIONS**	
1. Was the question for the review clearly focused and appropriate?	Yes No Unclear N/A
2. Was the search strategy used to locate relevant studies comprehensive? Were the databases searched and the search terms used described?	Yes No Unclear N/A
3. Were explicit methods used to select studies to include in the review? Were inclusion/exclusion criteria specified and appropriate? Were selection methods unbiased?	Yes No Unclear N/A
4. Was there an appraisal of the quality and validity of studies included in the review? Were appraisal methods specified, appropriate, and reproducible?	Yes No Unclear N/A
5. Were specific treatments/interventions/exposures described? Were treatments similar enough to be combined?	Yes No Unclear N/A
6. Was the outcome of interest clearly indicated? Were other potential harms and benefits considered?	Yes No Unclear N/A
7. Were processes for data abstraction, synthesis, and analysis described? Were they applied consistently across studies and groups? Was there appropriate use of qualitative and/or quantitative synthesis? Was variation in findings among studies analyzed? Were heterogeneity issued considered? If data from studies were aggregated for meta-analysis, was the procedure described?	Yes No Unclear N/A
8. Are the results clearly presented in narrative and/or quantitative terms? If summary statistics are used, are levels of significance and/or confidence intervals included?	Yes No Unclear N/A
9. Are conclusions supported by results with biases and limitations taken into consideration? Are limitations of the review identified and discussed?	Yes No Unclear N/A
10. Was bias due to the review's funding or sponsorship unlikely?	Yes No Unclear N/A
**MINUS/NEGATIVE (-)**<br>*If most (six or more) of the answers to the above validity questions are "No," the review should be designated with a minus (-) symbol on the Evidence Quality Worksheet.*	
**NEUTRAL (∅)**<br>*If the answer to any of the first four validity questions (1-4) is "No," but other criteria indicate strengths, the review should be designated with a neutral (∅) symbol on the Evidence Worksheet.*	

PLUS/POSITIVE (+)
If most of the answers to the above validity questions are "Yes" (must include criteria 1, 2, 3, and 4), the report should be designated with a plus symbol (+) on the Evidence Worksheet.

When these criteria for review articles are applied to narrative reviews from past years, it is practically impossible to get a positive rating. This is because authors seldom reported their search strategy and did not give explicit attention to the scientific quality of included research. Recent systematic reviews published in the peer reviewed literature may earn a positive (+) rating.

Instructions for Using the Quality Criteria Checklist

First, read carefully the research article. Then, while abstracting the key information onto the *Evidence Worksheet*, consider each of the relevance and validity questions on the *Quality Criteria Checklist* and answer a "yes" or "no" to each one. A record of the answers to each question is useful for checking work and verifying consistency among analysts (i.e., inter-rater reliability). The project manager, lead analyst and the expert work group will review and approve the abstracted worksheet and the checklist.

Sub-questions on the Quality Criteria Checklist: Primary Research identify points to consider when answering each Validity Question. Not all sub-questions are meant to apply in every study; and the yes/no determination is not based on adding up answers to sub-questions. A "yes" indicates that the criterion was adequately addressed in the report.

While all questions on the checklists are important to sound research, some criteria take on added importance in specific research designs. The Study Design, Distinguishing Characteristics, and Important Questions (found in Table 3.5), identifies sub-questions that are the most important consideration for each type of study. A well-planned and well-executed study would address these points, plus others, in the article.

Occasionally, a major question is not applicable (NA) to the specific study. Use of NA is indicated in relevance questions 1 and 4 and validity question 3 of the *Primary Research Checklist.*

Checklists include directions for assigning the overall designation (negative -, neutral ∅, or positive +). The determination is added to the appropriate item on the *Evidence Worksheet.*

Table 3.5 Study Design, Distinguishing Characteristics, and Important Considerations

Study design type	Distinguishing characteristics of design	Most important quality considerations (from checklist)*
**EXPERIMENTAL & QUASI-EXPERIMENTAL STUDIES**	**(Investigator manipulated independent variable, and a control group always used)**	
Randomized controlled trial	investigators manipulates	3.1, 3.2, 4.3

(Preferred for therapy and prevention questions)	treatment/intervention (independent variable)<br>randomization to groups	2.1, 2.3, 5.1, 5.2, 6.1, 6.3 – 6.7, 7.4
Nonrandomized trial<br><br>(Frequently used for therapy and prevention questions)	investigators manipulates treatment/intervention (independent variable)	2.1 – 2.3, 3.1 – 3.3, 4.3<br><br>5.1, 5.2, 6.1, 6.3 – 6.7, 7.1 – 7.7
**OBSERVATIONAL STUDIES**	**(Comparisons made)**	
Comparison of 2 or more groups<br>(also called prospective cohort)<br><br>(Preferred for etiology, causation, or harm questions)	comparison of existing "convenient" groups getting different interventions or exposures	2.1, 2.2, 4.3, 4.4, 7.1, 7.3, 7.4, 7.6, 7.7, 8.5<br><br>2.3, 3.2, 3.3, 5.2, 5.3, 6.2 – 6.7
Single group before-after or time series	subject serves as own control	2.1, 2.3, 2.4, 6.2, 7.4, 7.6<br>4.3, 5.1, 5.2, 6.3 – 6.7, 7.1 – 7.3, 7.5<br>3 – Not Applicable
Sensitivity & specificity of diagnostic test<br><br>(Preferred for diagnosis questions)	dichotomous (yes/no) outcome<br>comparison with "gold standard"	3.7, 4.5, 5.5<br><br>2.4, 6.8, 7.6
**EPIDEMIOLOGICAL ANALYTIC STUDIES**	**(Comparisons constructed analytically, groups created post hoc)**	
Cohort study<br><br>(Preferred for natural history and prognosis questions)	membership based on defining characteristic or factor	2.1, 4.3, 7.1, 7.3, 7.4, 7.7, 8.5<br><br>2.3, 3.4, 5.3, 6.3,
Case-control study<br><br>(Preferred for etiology, causation, or harm questions)	"cases" with outcome identified then "matched" with non-cases (controls) from same population<br>look back for exposure	2.1, 3.5, 4.3, 7.3, 7.4, 7.6, 7.7<br><br>2.3, 5.4, 6.3, 6.4
Cross-sectional study<br><br>(Preferred for diagnosis questions)<br>(Used for etiologic, causation, or harm questions)	outcome (dependent variable) and exposure (independent variable) measured at same time	4.3, 7.4, 7.7<br><br>2.1, 2.3, 2.4, 3.4, 5.3, 6.8, 7.2, 7.4 – 7.6<br>3 – Not Applicable, if comparison groups are not constructed

DESCRIPTIVE STUDIES	(No comparison)	
Case series	describe process and outcomes prospectively, "natural history" with no intervention	2.1, 4.3, 6.5, 6.6, 7.1, 7.4, 7.6<br>2.3, 2.4, 5.2, 5.3, 7.2, 7.3<br>3 – Not Applicable

The **bolded items** from the Quality Criteria Checklist: Primary Research are most important for that study design. The other (not bold) items are also common threats to validity in other study designs. For example, items 3.1, 3.2 and 4.3 from the Quality Criteria Checklist are most important if the study design is a randomized controlled trial, while in a cross-sectional study design, the most important items from the Quality Criteria Checklist are 4.3, 7.4 and 7.7.

Display all Checklists Relevant to a Particular Question in a Single Table

Because we are interested in the findings of many research studies as they relate to a particular question, the information from each Quality Criteria Checklist is combined into a single report. All checklists that are connected to worksheets linked to the same evidence analysis question are compiled into a Quality Criteria Summary. This table is linked to the evidence summary and is generated electronically after the analyst has completed the quality criteria checklist for each article (see Table 3.6).

The Summary allows members of the expert workgroup to quickly view answers to the questions in the Quality Criteria Checklist in a side-by-side comparison for each research study that is relevant to a particular question. This information will assist them when they make a determination about the grade or strength of the evidence available to answer the question.

Users of the evidence library can also view this information in the tabular format. The side-by-side comparison of constructs and domains for each research article may assist the user's understanding of the rationale for the overall grade assigned by the expert workgroup. Publishing the Summary online is another example of ADA's commitment to transparency.

Table 3.6 Example of a Quality Criteria Summary from Diabetes 1 and 2 EAL® Project

	Ash et al. 200	Berne et al 200!	Brinkworth et a	Brown SA, et al.	Derosa et al 20(	Hanefeld et al 2	Hollander PA, et	Kelley et al 200:	Li et al 2005	Manning et al 1!	Mayer-Davis et	McNulty et al 20	Metz et al 2000
Overall Quality Rating	+	+	+	+	+	+	+	+	Ø	+	+	+	+
**Relevance Questions**													
1. Would implementing the studied intervention or procedure (if found successful) result in improved outcomes for the patients/clients/population group? (Not Applicable for some epidemiological studies)	Yes	Yes	Yes		Yes	Yes	Yes	Yes	Yes	No	Yes	Yes	Yes
2. Did the authors study an outcome (dependent variable) or topic that the patients/clients/population group would care about?	Yes	Yes	Yes		Yes	Yes	Yes	Yes	Yes	Yes	Yes	Yes	Yes
3. Is the focus of the intervention or procedure (independent variable) or topic of study a common issue of concern to dieteticspractice?	Yes	Yes	Yes		Yes	Yes	Yes	Yes	Yes	Yes	Yes	Yes	Yes
4. Is the intervention or procedure feasible? (NA for some epidemiological studies)	Yes	Yes	Yes		Yes	Yes	Yes	Yes	Yes	No	Yes	Yes	Yes
**Validity Questions**													
1. Was the research question clearly stated?	Yes	Yes	Yes		Yes	Yes	Yes	Yes	Yes	Yes	Yes	Yes	Yes
2. Was the selection of study subjects/patients free from bias?	Yes	Yes	Yes		Yes	Yes	Yes	Yes	Yes	Yes	Yes	Yes	Yes
3. Were study groups comparable?	Yes	Yes	Yes		Yes	Yes	Yes	Yes	???	Yes	Yes	Yes	Yes
4. Was method of handling withdrawals described?	Yes	Yes	Yes		Yes	Yes	Yes	Yes	Yes	Yes	Yes	Yes	Yes
5. Was blinding used to prevent introduction of bias?	Yes	Yes	No		Yes	Yes	Yes	Yes	Yes	???	Yes	Yes	Yes
6. Were intervention/therapeutic regimens/exposure factor or procedure and any comparison(s) described in detail? Were interveningfactors described?	Yes	Yes	Yes		Yes	Yes	Yes	Yes	Yes	Yes	Yes	Yes	Yes
7. Were outcomes clearly defined and the measurements valid and reliable?	Yes	Yes	Yes		Yes	Yes	Yes	Yes	Yes	Yes	Yes	Yes	Yes

At the end of Step 3 the following materials are available for each question on the Portal. The expert panel can review these items on the Preview site.

- The Question
- *Sort List / Search Plan* & *Results*
- Full text of each article
- Abstracted Worksheets for each article
- Quality Criteria Checklists for each article
- Quality Criteria Summary combining all checklists

Appendices

Appendix 7: Evidence Abstract Worksheet Template

<table>
<tr><td>Citation:</td><td></td></tr>
<tr><td>Study Design:</td><td></td></tr>
<tr><td>Class:</td><td>Based on classes of evidence reports (Table 2.3)</td></tr>
<tr><td>Quality Rating:</td><td>+, Ø , - Based on Quality Criteria Checklist</td></tr>
<tr><td>Research Purpose:</td><td></td></tr>
<tr><td>Inclusion Criteria:</td><td></td></tr>
<tr><td>Exclusion Criteria:</td><td></td></tr>
<tr><td>Description of Study Protocol:</td><td>Recruitment
Design
Blinding used (if applicable)
Intervention (if applicable)
Statistical Analysis
(These prompts assist you in determining which information to abstract from research article.)</td></tr>
<tr><td>Data Collection Summary:</td><td>Timing of Measurements
Dependent Variables
• Variable 1: brief description (how measured?)
• Variable 2: brief description (how measured?)
• etc.
Independent Variables
Control Variables</td></tr>
<tr><td>Description of Actual Data Sample:</td><td>Initial N: (e.g., 731 (298 males, 433 females))
Attrition (final N):
Age:
Ethnicity:
Other relevant demographics:
Anthropometrics (e.g., were groups same or different on important measures)
Location:</td></tr>
<tr><td>Summary of Results:</td><td>
<table>
<tr><td colspan="4">Key Findings</td></tr>
<tr><td>Variables</td><td>Treatment Group Measures and confidence intervals</td><td>Control group Measures and confidence intervals</td><td>Statistical Significance of Group Difference</td></tr>
<tr><td>Dep var 1</td><td>Mean, CI.
e.g., 4.5±2.2</td><td>Mean, CI.
e.g., 1.5±2.0</td><td>Stat signif difference between groups
e.g., p=.002</td></tr>
<tr><td>Dep var 2</td><td></td><td></td><td></td></tr>
</table>
</td></tr>
</table>

	Etc. | | | **Other Findings**
**Author Conclusion:**	
**Review Comments:**	*Italicize reviewer and expert panel comments.*
**Funding Source**	Determine the funding source: Government, Industry, University/Hospital , Not-for-Profit and/or Other.

Appendix 8: Quality Criteria Checklist: Primary Research

Symbols Used

+ **Positive:** Indicates that the report has clearly addressed issues of inclusion/exclusion, bias, generalizability, and data collection and analysis.

-- **Negative:** Indicates that these issues have not been adequately addressed.

∅ **Neutral:** Indicates that the report is neither exceptionally strong nor exceptionally weak.

Quality Criteria Checklist: Primary Research

RELEVANCE QUESTIONS	
5. Would implementing the studied intervention or procedure (if found successful) result in improved outcomes for the patients/clients/population group? (NA for some Epi studies)	Yes No Unclear N/A
6. Did the authors study an outcome (dependent variable) or topic that the patients/clients/population group would care about?	Yes No Unclear N/A
7. Is the focus of the intervention or procedure (independent variable) or topic of study a common issue of concern to dietetics practice?	Yes No Unclear N/A
8. Is the intervention or procedure feasible? (NA for some epidemiological studies)	Yes No Unclear N/A
If the answers to all of the above relevance questions are "Yes," the report is eligible for designation with a plus (+) on the Evidence Quality Worksheet, depending on answers to the following validity questions.	
**VALIDITY QUESTIONS**	
**11. Was the <u>research question</u> clearly stated?**<br>1.1 Was the specific intervention(s) or procedure (independent variable(s)) identified?<br>1.2 Was the outcome(s) (dependent variable(s)) clearly indicated?<br>1.3 Were the target population and setting specified?	Yes No Unclear N/A
**12. Was the <u>selection</u> of study subjects/patients free from bias?**<br>2.1 Were inclusion/exclusion criteria specified (e.g., risk, point in disease progression, diagnostic or prognosis criteria), and with sufficient detail and without omitting criteria critical to the study?<br>2.2 Were criteria applied equally to all study groups?<br>2.3 Were health, demographics, and other characteristics of subjects described?<br>2.4 Were the subjects/patients a representative sample of the relevant population?	Yes No Unclear N/A
**13. Were <u>study groups comparable</u>?**<br>3.1 Was the method of assigning subjects/patients to groups described and unbiased? (Method of randomization identified if RCT)<br>3.2 Were distribution of disease status, prognostic factors, and other factors (e.g., demographics) similar across study groups at baseline?<br>3.3 Were concurrent controls used? (Concurrent preferred over historical controls.)<br>3.4 If cohort study or cross-sectional study, were groups comparable on important confounding factors and/or were preexisting differences accounted for by using appropriate adjustments in statistical analysis?<br>3.5 If case control study, were potential confounding factors comparable for cases and controls? (If case series or trial with subjects serving as own control, this criterion is not applicable. Criterion may not be applicable in some cross-sectional studies.)<br>3.6 If diagnostic test, was there an independent blind comparison with an appropriate reference standard (e.g., "gold standard")?	Yes No Unclear N/A
**14. Was method of handling <u>withdrawals</u> described?**<br>4.1 Were follow up methods described and the same for all groups?<br>4.2 Was the number, characteristics of withdrawals (i.e., dropouts, lost to follow up, attrition rate) and/or response rate (cross-sectional studies) described for each group? (Follow up goal for a strong study is 80%.)<br>4.3 Were all enrolled subjects/patients (in the original sample) accounted for?<br>4.4 Were reasons for withdrawals similar across groups?	Yes No Unclear N/A

4.5 If diagnostic test, was decision to perform reference test not dependent on results of test under study?	
**15. Was <u>blinding</u> used to prevent introduction of bias?**	Yes No Unclear N/A
5.1 In intervention study, were subjects, clinicians/practitioners, and investigators blinded to treatment group, as appropriate?	
5.2 Were data collectors blinded for outcomes assessment? (If outcome is measured using an objective test, such as a lab value, this criterion is assumed to be met.)	
5.3 In cohort study or cross-sectional study, were measurements of outcomes and risk factors blinded?	
5.4 In case control study, was case definition explicit and case ascertainment not influenced by exposure status?	
5.5 In diagnostic study, were test results blinded to patient history and other test results?	
**16. Were <u>intervention</u>/therapeutic regimens/exposure factor or procedure and any comparison(s) described in detail? Were <u>intervening factors</u> described?**	Yes No Unclear N/A
6.1 In RCT or other intervention trial, were protocols described for all regimens studied?	
6.2 n observational study, were interventions, study settings, and clinicians/provider described?	
6.3 Was the intensity and duration of the intervention or exposure factor sufficient to produce a meaningful effect?	
6.4 Was the amount of exposure and, if relevant, subject/patient compliance measured?	
6.5 Were co-interventions (e.g., ancillary treatments, other therapies) described?	
6.6 Were extra or unplanned treatments described?	
6.7 Was the information for 6.4, 6.5, and 6.6 assessed the same way for all groups?	
6.8 In diagnostic study, were details of test administration and replication sufficient?	
**17. Were <u>outcomes</u> clearly defined and the <u>measurements valid and reliable</u>?**	Yes No Unclear N/A
7.1 Were primary and secondary endpoints described and relevant to the question?	
7.2 Were nutrition measures appropriate to question and outcomes of concern?	
7.3 Was the period of follow-up long enough for important outcome(s) to occur?	
7.4 Were the observations and measurements based on standard, valid, and reliable data collection instruments/tests/procedures?	
7.5 Was the measurement of effect at an appropriate level of precision?	
7.6 Were other factors accounted for (measured) that could affect outcomes?	
7.7 Were the measurements conducted consistently across groups?	
**18. Was the <u>statistical analysis</u> appropriate for the study design and type of outcome indicators?**	Yes No Unclear N/A
8.1 Were statistical analyses adequately described the results reported appropriately?	
8.2 Were correct statistical tests used and assumptions of test not violated?	
8.3 Were statistics reported with levels of significance and/or confidence intervals?	
8.4 Was “intent to treat” analysis of outcomes done (and as appropriate, was there an analysis of outcomes for those maximally exposed or a dose-response analysis)?	
8.5 Were adequate adjustments made for effects of confounding factors that might have affected the outcomes (e.g., multivariate analyses)?	
8.6 Was clinical significance as well as statistical significance reported?	
8.7 If negative findings, was a power calculation reported to address type 2 error?	
**19. Are <u>conclusions supported by results</u> with biases and limitations taken into consideration?**	Yes No Unclear N/A
9.1 Is there a discussion of findings?	
9.2 Are biases and study limitations identified and discussed?	
**20. Is bias due to study’s <u>funding or sponsorship</u> unlikely?**	Yes No Unclear N/A
10.1 Were sources of funding and investigators’ affiliations described?	
10.2 Was there no apparent conflict of interest?	
**MINUS/NEGATIVE (-)** *If most (six or more) of the answers to the above validity questions are “No,” the report should be designated with a minus (-) symbol on the Evidence Worksheet.*	

**NEUTRAL (∅)**
If the answers to validity criteria questions 2, 3, 6, and 7 do not indicate that the study is exceptionally strong, the report should be designated with a neutral (∅) symbol on the Evidence Worksheet.
**PLUS/POSITIVE (+)**
If most of the answers to the above validity questions are "Yes" (including criteria 2, 3, 6, 7 and at least one additional "Yes"), the report should be designated with a plus symbol (+) on the Evidence Worksheet.

Appendix 9: Quality Criteria Checklist: Review Article

Symbols Used

+ **Positive:** Indicates that the report has clearly addressed issues of inclusion/exclusion, bias, generalizability, and data collection and analysis.

-- **Negative:** Indicates that these issues have not been adequately addressed.

∅ **Neutral:** Indicates that the report is neither exceptionally strong nor exceptionally weak.

Quality Criteria Checklist: Review Articles

RELEVANCE QUESTIONS	
5. Will the answer if true, have a direct bearing on the health of patients?	Yes No Unclear N/A
6. Is the outcome or topic something that patients/clients/population groups would care about?	Yes No Unclear N/A
7. Is the problem addressed in the review one that is relevant to dietetics practice?	Yes No Unclear N/A
8. Will the information, if true, require a change in practice?	Yes No Unclear N/A
If the answers to all of the above relevance questions are "Yes," the report is eligible for designation with a plus (+) on the Evidence Quality Worksheet, depending on answers to the following validity questions.	
**VALIDITY QUESTIONS**	
11. Was the question for the review clearly focused and appropriate?	Yes No Unclear N/A
12. Was the search strategy used to locate relevant studies comprehensive? Were the databases searched and the search terms used described?	Yes No Unclear N/A
13. Were explicit methods used to select studies to include in the review? Were inclusion/exclusion criteria specified and appropriate? Were selection methods unbiased?	Yes No Unclear N/A
14. Was there an appraisal of the quality and validity of studies included in the review? Were appraisal methods specified, appropriate, and reproducible?	Yes No Unclear N/A
15. Were specific treatments/interventions/exposures described? Were treatments similar enough to be combined?	Yes No Unclear N/A
16. Was the outcome of interest clearly indicated? Were other potential harms and benefits considered?	Yes No Unclear N/A
17. Were processes for data abstraction, synthesis, and analysis described? Were they applied consistently across studies and groups? Was there appropriate use of qualitative and/or quantitative synthesis? Was variation in findings among studies analyzed? Were heterogeneity issued considered? If data from studies were aggregated for meta-analysis, was the procedure described?	Yes No Unclear N/A
18. Are the results clearly presented in narrative and/or quantitative terms? If summary statistics are used, are levels of significance and/or confidence intervals included?	Yes No Unclear N/A
19. Are conclusions supported by results with biases and limitations taken into consideration? Are limitations of the review identified and discussed?	Yes No Unclear N/A
20. Was bias due to the review's funding or sponsorship unlikely?	Yes No Unclear N/A
**MINUS/NEGATIVE (-)**<br>*If most (six or more) of the answers to the above validity questions are "No," the review should be designated with a minus (-) symbol on the Evidence Quality Worksheet.*	
**NEUTRAL (∅)**<br>*If the answer to any of the first four validity questions (1-4) is "No," but other criteria indicate strengths, the review should be designated with a neutral (∅) symbol on the Evidence Worksheet.*	
**PLUS/POSITIVE (+)**<br>*If most of the answers to the above validity questions are "Yes" (must include criteria 1, 2, 3, and 4), the report should be designated with a plus symbol (+) on the Evidence Worksheet.*	

Appendix 10: Important Considerations from Checklist by Study Design

Study design type	Distinguishing characteristics of design	Most important considerations (from checklist)*
**EXPERIMENTAL & QUASI-EXPERIMENTAL STUDIES**	**(Investigator manipulated independent variable always control group)**	
Randomized controlled trial<br><br>(Preferred for therapy and prevention questions)	investigators manipulates treatment/intervention (independent variable)<br>randomization to groups	3.1, 3.2, 4.3<br><br>2.1, 2.3, 5.1, 5.2, 6.1, 6.3 – 6.7, 7.4
Nonrandomized trial<br><br>(Frequently used for therapy and prevention questions)	investigators manipulates treatment/intervention (independent variable)	2.1, 2.3, 3.1-3.3, 4.3<br><br>5.1, 5.2, 6.1, 6.3 – 6.7, 7.1 – 7.7
**OBSERVATIONAL STUDIES**	**(Comparisons made)**	
Comparison of 2 or more groups<br>(also called prospective cohort)<br><br>(Preferred for etiology, causation, or harm questions)	comparison of existing "convenient" groups getting different interventions or exposures	2.1, 2.2, 4.3, 4.4, 7.1, 7.3, 7.4, 7.6, 7.7, 8.5<br><br>2.3, 3.2, 3.3, 5.2, 5.3, 6.2 – 6.7
Single group before-after or time series	subject serves as own control	2.1, 2.3, 2.4, 6.2, 7.4, 7.6<br>4.3, 5.1, 5.2, 6.3 – 6.7, 7.1 – 7.3, 7.5<br>3 - NA**
Sensitivity & specificity of diagnostic test<br><br>(Preferred for diagnosis questions)	dichotomous (yes/no) outcome<br>comparison with "gold standard"	3g, 4e, 5e<br><br>2.4, 6.8, 7.6
**EPIDEMIOLOGICAL ANALYTIC STUDIES**	**(Comparisons constructed analytically, groups created post hoc)**	
Cohort study<br><br>(Preferred for natural history and prognosis questions)	membership based on defining characteristic or factor	2.1, 4.3, 7.1, 7.3, 7.4, 7.6, 8.5<br><br>2.3, 3.4, 5.3, 6.3
Case-control study<br><br>(Preferred for etiology, causation, or harm questions)	"cases" with outcome identified then "matched" with non-cases (controls) from same population<br>look back for exposure	2.1, 3.5, 4.3, 7.3, 7.4 7.6, 7.7<br><br>2.3, 5.4, 6.3, 6.4
Cross-sectional study<br><br>(Preferred for diagnosis questions)<br>(Used for etiologic, causation, or harm questions)	outcome (dependent variable) and exposure (independent variable) measured at same time	4.3, 7.4, 7.6<br><br>2.1, 2.3, 2.4, 3.4, 5.3, 6.8, 7.2, 7.4 – 7.6<br>3 - NA, if comparison groups are not constructed
**DESCRIPTIVE STUDIES**	**(No comparison)**	
Case series	describe process and outcomes prospectively, "natural history" with no intervention	2.1, 4.3, 6.5, 6.6, 7.1, 7.4, 7.6<br>2.3, 2.4, 5.2, 5.3, 7.2, 7.3<br>3 - NA

*See: *Quality Criteria Checklist*: Primary Research. Bolded items are most important for study design. The other (not bold) items are also common threats to validity in study type.

**NA = not applicable

Appendix 11: Tally Sheet of Quality Criteria Ratings

	Author A	Author B	Author C	Author D
Year				
Relevance Questions				
1				
2				
3				
4				
Validity Questions				
1				
2				
3				
4				
5				
6				
7				
8				
9				
10				
Quality Rating (+,0,-)				

For each question, a table should be created to combine the answers from the quality criteria checklists completed for each article. The ADA online tool will generate this table for each question from the completed checklists and make this tally available to all users of the ADA Evidence Analysis Library®.

Appendix12: Sample Tally Sheet from the EAL®

	Ash et al, 2003	Berne et al 200	Brinkworth et a	Brown SA, et al.	Derosa et al 20	Hanefeld et al 2	Hollander PA, et	Kelley et al 200	Li et al 2005	Manning et al 1	Mayer-Davis et	McNulty et al 20	Metz et al 2000
Overall Quality Rating	+	+	+	+	+	+	+	+	○	+	+	+	+
**Relevance Questions**													
1. Would implementing the studied intervention or procedure (if found successful) result in improved outcomes for the patients/clients/population group? (Not Applicable for some epidemiological studies)	Yes	Yes	Yes		Yes	Yes	Yes	Yes	Yes	No	Yes	Yes	Yes
2. Did the authors study an outcome (dependent variable) or topic that the patients/clients/population group would care about?	Yes	Yes	Yes		Yes	Yes	Yes	Yes	Yes	Yes	Yes	Yes	Yes
3. Is the focus of the intervention or procedure (independent variable) or topic of study a common issue of concern to dieteticspractice?	Yes	Yes	Yes		Yes	Yes	Yes	Yes	Yes	Yes	Yes	Yes	Yes
4. Is the intervention or procedure feasible? (NA for some epidemiological studies)	Yes	Yes	Yes		Yes	Yes	Yes	Yes	Yes	No	Yes	Yes	Yes
**Validity Questions**													
1. Was the research question clearly stated?	Yes	Yes	Yes		Yes	Yes	Yes	Yes	Yes	Yes	Yes	Yes	Yes
2. Was the selection of study subjects/patients free from bias?	Yes	Yes	Yes		Yes	Yes	Yes	Yes	Yes	Yes	Yes	Yes	Yes
3. Were study groups comparable?	Yes	Yes	Yes		Yes	Yes	Yes	Yes	???	Yes	Yes	Yes	Yes
4. Was method of handling withdrawals described?	Yes	Yes	Yes		Yes	Yes	Yes	Yes	Yes	Yes	Yes	Yes	Yes
5. Was blinding used to prevent introduction of bias?	Yes	Yes	No		Yes	Yes	Yes	Yes	Yes	???	Yes	Yes	Yes
6. Were intervention/therapeutic regimens/exposure factor or procedure and any comparison(s) described in detail? Were interveningfactors described?	Yes	Yes	Yes		Yes	Yes	Yes	Yes	Yes	Yes	Yes	Yes	Yes
7. Were outcomes clearly defined and the measurements valid and reliable?	Yes	Yes	Yes		Yes	Yes	Yes	Yes	Yes	Yes	Yes	Yes	Yes
