# Supplementary material for: Funding Source and Research Report Quality in Nutrition Practice-Related Research
Source: PLoS One. 2011 Dec 6;6(12):e28437. doi: 10.1371/journal.pone.0028437 (PMC3232225; doi:10.1371/journal.pone.0028437)
Supplement: Supporting Information S2 — Summary of Topics and Keywords from the Evidence Analysis Library database on February 2009 (expansion of Figure 5 ). (DOC) [file pone.0028437.s002.doc]

**Supporting Information 2.** Description of topics and keywords included in library database^1^ and published on the ADA Evidence Analysis Library prior to March 2009

| **Topic** | | **Number of PICO questions** | **# of Research Reports (abstracted articles/worksheets)^2^** | **Keywords** | |
| --- | --- | --- | --- | --- | --- |
| Adult Weight Management | | 16 questions | 209 | Weight loss, maintenance, cognitive restructuring, self-monitoring, food guide pyramid, healthy food choices, low carbohydrate diets, regular meal and snack pattern, meal replacements, high calcium, dairy, portion size, low glycemic index foods, client adherence, eating breakfast, healthy cooking techniques, recipe modification, improved eating habits, reading nutrition information and food labels, reducing fat mass, Very Low Calorie Diet, Low Calorie Diet, long-term maintenance | |
| Breastfeeding | | 3 questions | 38 | Breast milk production, lactation, artificial nipple, duration of breastfeeding, healthy term, full term infants, pregnant, maternal diet, dietary supplements, omega-3 fatty acid, breast milk composition, infant health outcomes | |
| Childhood Overweight | | 34 questions | 227 | Breakfast skipping, adiposity, intake of dairy, eating frequency, eating out, household food insecurity, childhood overweight, 100% fruit juice, fruits, vegetables, portion size, snacking, calorically-sweetened beverages, family functioning, overweight, parental attitudes; dietary restraint; pressure to eat; parental control, child dietary intake, highly palatable foods, food as a reward, emotional feeding, television viewing, video games, physical activity, sports, exercise programs, dietary fat, total energy intake, school-based programs, intervention, media influences, nutrition education, school meals, school foods, sedentary activity, homework, reading, school-based intervention, feeding strategies, calcium intake | |
| Chronic Kidney Disease | | 5 questions | 18 | Physical activity interventions, disease progression, quality of life, fish oil therapy, IgA nephropathy, kidney transplant, rental transplant, nutritional needs, wound treatment modalities, debridement, cleansing, dressing, chronic kidney disease | |
| Chronic Obstructive Pulmonary Disease (COPD) | | 10 questions | 84 | Energy needs in patients with COPD, ambulatory, consumption of milk, mucus production, body weight, dietary intake, quality of life, steroids, bone mineral density, antioxidants, vitamin A, vitamin C, vitamin E, flavonoids, pathogenesis, weight loss, nutritional supplementation, risk factors of osteoporosis, supplemental oxygen | |
| Critical Illness | | 83 questions | 135 | Enteral nutrition versus parenteral nutrition, infectious complications, cost of medical care, length of hospital stay, enteral feeding tube tip, gastric, small bowel position, LOS, timing of enteral feeding, mortality, blue dye, aspiration, gastric volume level, gastric residual volume, pneumonia, head elevation, reflux, immune-modulating, nutrient composition, blood glucose control, mortality, determination of resting metabolic rate, RMR, Harris-Benedict equation, Ireton-Jones 1992 equation, Ireton-Jones 1997 equation, Penn State equation, Mifflin-St. Jeor equation, Swinamer equation, elevated BMI, low BMI, weight, trauma, cardiovascular, surgical, energy intake, protein intake, protein delivery, caloric delivery, rate of infection complications, mechanical ventilation | |
| Diabetes 1 and 2 | | 16 questions | 225 | Self-monitoring; blood glucose, metabolic outcomes, type 1, type 2, diabetes, protein intake, weight management, physical activity, MNT, carbohydrate intake, interventions, cardiovascular disease, nutrition interventions, protein restriction, amino acid, ketoacid supplementation, diabetic nephropathy, fiber, glycemic index | |
| Disorders of Lipid Metabolism (DLM) | | 77 questions | 239 | Consuming nuts, cholesterol levels, risk of CHD, fish-derived omega-3, fatty acids, cardiac death, angina, plant-derived, hypertension, fiber intake, coronary heart disease, high fiber foods, saturated fat, total cholesterol, LDL, soluble fiber, plant stanols, sterols, esterified stanols, nonesterfied stanols, statin, carotenoid-rich fruits, soy protein, serum total cholesterol, triglycerdies levels, isoflavones, vitamin E, beta0-carotene, vitamin C, selenium, simvastatin-niacin, antioxidants, lipid levels, coenzyme Q10, beta-carotene, antioxidants, serum homocysteine, supplemental folate, vitamin B6, vitamin B12, trans fatty acids, MNT, registered dietitian, RD, HMG-CoA reductase inhibitors, drinking alcohol, alcoholic beverages, lipid lowering medications, BMI, wait circumference, waist-to-hip ratio, abdominal obesity, exercise, metabolic syndrome, EPA/DHA, fish oil supplement, hypertriglycerdemic patients, DHA-enriched eggs, almonds, portfolio diet, | |
| Energy Expenditure | | 146 questions | 192 | Resting metabolic rate, RMR, circulatory hormone changes, hormone replacement therapy, HRT, birth control medications, RQ, alcohol, caffeine, obese, Japanese women, smokers, chemical use, chronic thermic effects of 7-keto-Naturalean, ephedra, nicotine, cessation of nicotine, indirect calorimetry measurement, ventilated patients, energy measurement, Veterans Health Administration, acute care, trauma setting, non-white ethnic groups, oxygen consumption, carbon dioxide production, coefficient variation, medically stable institutionalized, respiratory quotient, respiratory conditions, humidity control, noise control, light control, postmenopausal, obese women, temperature control, gas collection devices, facemask, mouthpieces, walking, jogging, cycling, resistance exercise, thyroid-stimulating hormone, thyroxine, triiodothyronine, contraception, estrogen, estradiol, progesterone, premenopausal, Polycystic Ovarian Syndrome, PCOS, overestimation, underestimation, Asian women, Harris-Benedict formula, Owen et al formula, Mifflin-St. Jeor formula, estimating resting metabolic rate, group mean errors, prediction accuracy, moderate cold, ambient temperature, semi-recumbent, supine positions, Thermic Effect of Food, TEF, meal peaks | |
| Fiber | | 4 questions | 50 | Dietary fiber, whole foods, supplements, diabetes, obesity, gastrointestinal health and disease, cardiovascular disease | |
| Nutrition Supplementation | | 7 questions | 44 | Oral vitamin B12, serum cobalamin levels, older adults, cognitive function, 25 hydroxyvitamin D, bone health outcomes, post-menopausal women, older adult men, vitamin D, bone density, fractures, falls | |
| Gestational Diabetes | | 13 questions | 125 | Gestational Diabetes Mellitus, exercise, physical activity, pregnancy, neonatal outcomes, glycemic control, prevention, nutrition interventions, caloric restriction, weight management, ketonuria, medical nutrition therapy, pregnancy outcomes, morbidity, birth weight, glucose control, pharmacological therapy, pre-term delivery, satisfaction with care; carbohydrate, fat, protein, type 2 diabetes, breastfeeding, blood glucose monitoring, ketone testing, nonnutritive sweeteners, impaired glucose tolerance, caloric intake recommendation, appropriate weight gain | |
| Celiac Disease | | 12 questions | 112 | Celiac Disease, oats, wheat, Codex Alimentarius, iron deficiency anemia, bone density, villous atrophy, pregnancy, neurological symptoms, effectiveness of dietary pattern, gastrointestinal symptoms, quality of life, nutritional adequacy | |
| Heart Failure | | 16 questions | 35 | Heart failure, L-arginine, carnitine, hawthorn, CoEnzyme Q10, thiamine, potassium, magnesium, folate, vitamin B12, alcohol intake, sodium, morbidity, mortality, fluid, medical nutrition therapy, omega-3 fatty acids, lean body mass, BMI, underweight, overweight, caffeine | |
| HIV/AIDS | | 4 questions | 33 | HIV infection, energy; dietary fatty acids, protein, carbohydrate, dietary intake | |
| Hydration | | 8 questions | 77 | Caffeinated beverages, fluids, thickened beverages, fluid intake, hydration, hydration status, biochemical parameter, electrolytes, physical activity, exercise, estimate fluid requirements, continuous endurance, restore hydration, dysphagia, | |
| Hypertension | | 15 questions | 100 | Magnesium intake, blood pressure, healthy, hypertensive, potassium intake, caffeine intake, protein intake, omega-3 fatty acid, vitamin C, vitamin E, garlic intake, soluble fiber, cocoa, chocolate intake, calcium, sodium, dairy protein , soy food intake, fruit and vegetable intake | |
| Medical Nutrition Therapy Effectiveness | | 5 questions | 31 | Medical nutrition therapy, MNT, cost-effectiveness, cost benefit, economic savings, inpatient, services provided, RD, registered dietitian, healthcare professionals, nutrition interventions, outpatient, counseling, healthcare team, lifestyle interventions for diabetes prevention | |
| Non-nutritive sweeteners | | 12 questions | 65 | Non-nutritive sweeteners, FDA, saccharin, aspartame, acesulfame-K, sucralose, neotame, consumption levels, energy density, appetite, diabetes, food intake in children, daily intake limits, children, pregnant women | |
| Nutrition Counseling | | 25 questions | 81 | Cognitive-behavioral therapy, short-term duration, weight loss, social learning theory, cardiovascular disease, diabetic management, intermediate duration, health/food behavior change; prevention or delayed onset of diabetes, social learning theory, transtheoretical model, adults counseled, outpatient, nutrition counseling, motivational interviewing, counseled, self-monitoring, behavioral program, group-based nutrition counseling, behavioral strategy, problem-solving, meal replacements, structured meal plans, reward, reinforcement, contingency management, social support, goal-setting, long-term duration, weight management, long-term maintenance | |
| Nutrition in Athletic Performance | | 5 questions | 111 | Meal timing, coloric intake, macronutrient intake, optimal athletic performance, training, energy balance, energy imbalance, body composition, eight management, meal timing, competition, | |
| Nutrition Screening | | 2 questions | 1 | Validity, Simple Two-part Screening Tool, nutrition problems, acute care, hospital-based ambulatory care setting, Short Nutritional Assessment Questionnaire, SNQ, nutrition problems | |
| Oncology | | 111 questions | 120 | Cancer, food texture to reduce symptoms, patient’s alteration, food temperature, food acidity, food seasoning, soluble fiber, prostate cancer, low-residue diet, pancreatic cancer, glutamine intake, arginine-enhanced medical food supplement, plasma protein, post-surgical, head and neck cancer, oral intake, BCAA, liver cancer, probiotics, honey intake, ginger intake, anticancer therapy, chemotherapy, radiation therapy, improve tolerance, hot temperature foods, oral mucositis, spicy foods, radiation-induced diarrhea, pelvic radiation, gynecological cancer, postoperative patients, enteral nutrition, immune function, omega-3 fatty acids, RNA-containing nutritional supplement, preoperative,, HMB, hematopoietic cell transplantation HCT, brain cancer, fish oil supplement, weight loss, oral granular, chemoembolization, amino acids, vitamin E, breast cancer, oral cancer, laryngeal cancer, interleukin therapy IL-2, parenteral nutrition, vitamin C, medical food supplements, selenium, cisplatin-based chemotherapy, palitaxel chemotherapy, carboplatin chemotherapy, graft-versus –host-disease, allogeneic hematopoietic cell transplant, malignancy, lipid-based TPN, catabolic process, lung cancer, non-small lung cancer, energy needs, testicular cance3r, melanoma, rectal cancer, protein requirements, | |
| Pediatric Weight Management | | 52 questions | 66 | Prescribed dietary plan, intervention program, child, obesity, ages 6-12, family-based counseling, children, parent training, pediatric weight management, psychotherapy, multicomponent, adolescent, peer modeling, dietary component, physical activity, sedentary behavior, behavioral counseling, food guide pyramid, limit calorie intake, limit food intake, traffic light diet, low-glycemic diet, low fat diet, libitum, low carbohydrate diet, protein sparing, fast diets, weight loss, daily energy intake, body mass, growth velocity, protein sparing modified fast diet, child-only treatment, adolescent-only treatment, weight loss, parent, low calorie, balanced macronutrient, low calorie, orlistat, sibutramine, group sessions, individual session | |
| Sodium | | 3 questions | 6 | Sodium, blood pressure, Hispanics, hypertension, children, sodium intake | |
| Spinal Cord Injury | | 12 questions | 67 | Lipid abnormalities, spinal cord injury, cranberry juice, urologic health, urinary track infections, stone formation, acute care, rehabilitation, community settings, caloric needs, body composition, fiber, neurogenic bowel, bowel, bladder, pressure ulcers, physical activity, energy needs, protein needs, overweight, obesity, cranberry extract supplements, | |
| Unintended Weight Loss | | 5 questions | 22 | Modified texture diets, weight gain, adults, over age 65, caloric needs, healthy older adults, acutely underweight, chronically ill, underweight, activity level, measured RMR | |
| Vegetarian Nutrition | | 29 questions | 59 | Macronutrient, energy intake, pregnant vegetarians, pregnant omnivores, bioavailability, vegan, birth outcomes, omnivorous diet, ischemic heart disease, vegetarian diet, micronutrient intake, macronutrient intake, adults, children, therapeutic use, obese, overweight, triglyceride levels, cholesterol, LDL cholesterol, HDL cholesterol, VLDL, blood glucose, diabetes, A1C level, insulin levels, therapeutic use of vegetarian diet | |
| **29 Topics** | | **732 questions** | **2573 ^2^** |  | |
|  |  | | | |  |
|  |  | | | |  |

^1^The ADA Evidence Analysis Library database contained 29 topics, 732 questions and 2539 unique abstracted research reports (worksheets) with quality checklists at the time of this study.

^2^Some worksheets were used in more than one project.
